# Hypoxic Regulation of *Hand1* Controls the Fetal-Neonatal Switch in Cardiac Metabolism

**DOI:** 10.1371/journal.pbio.1001666

**Published:** 2013-09-24

**Authors:** Ross A. Breckenridge, Izabela Piotrowska, Keat-Eng Ng, Timothy J. Ragan, James A. West, Surendra Kotecha, Norma Towers, Michael Bennett, Petra C. Kienesberger, Ryszard T. Smolenski, Hillary K. Siddall, John L. Offer, Mihaela M. Mocanu, Derek M. Yelon, Jason R. B. Dyck, Jules L. Griffin, Andrey Y. Abramov, Alex P. Gould, Timothy J. Mohun

**Affiliations:** 1 Developmental Biology, MRC–National Institute for Medical Research, London, United Kingdom; 2 Division of Medicine, University College London, London, United Kingdom; 3 Division of Molecular Structure, MRC–National Institute for Medical Research, London, United Kingdom; 4 Department of Biochemistry, Cambridge University, Cambridge, United Kingdom; 5 Cardiovascular Research Centre, Faculty of Medicine and Dentistry, University of Alberta, Edmonton, Alberta, Canada; 6 Department of Biochemistry, Medical University of Gdansk, Poland; 7 Hatter Institute, Institute of Cardiovascular Sciences, University College London, London, United Kingdom; 8 Physical Biochemistry, MRC–National Institute for Medical Research, London, United Kingdom; 9 Institute of Neurology, University College London, London, United Kingdom; 10 Division of Physiology and Metabolism, MRC–National Institute for Medical Research, London, United Kingdom; University of Washington, United States of America

## Abstract

Cardiomyocytes are vulnerable to hypoxia in the adult, but adapted to hypoxia *in utero*. Current understanding of endogenous cardiac oxygen sensing pathways is limited. Myocardial oxygen consumption is determined by regulation of energy metabolism, which shifts from glycolysis to lipid oxidation soon after birth, and is reversed in failing adult hearts, accompanying re-expression of several “fetal” genes whose role in disease phenotypes remains unknown. Here we show that hypoxia-controlled expression of the transcription factor Hand1 determines oxygen consumption by inhibition of lipid metabolism in the fetal and adult cardiomyocyte, leading to downregulation of mitochondrial energy generation. Hand1 is under direct transcriptional control by HIF1α. Transgenic mice prolonging cardiac Hand1 expression die immediately following birth, failing to activate the neonatal lipid metabolising gene expression programme. Deletion of Hand1 in embryonic cardiomyocytes results in premature expression of these genes. Using metabolic flux analysis, we show that Hand1 expression controls cardiomyocyte oxygen consumption by direct transcriptional repression of lipid metabolising genes. This leads, in turn, to increased production of lactate from glucose, decreased lipid oxidation, reduced inner mitochondrial membrane potential, and mitochondrial ATP generation. We found that this pathway is active in adult cardiomyocytes. Up-regulation of Hand1 is protective in a mouse model of myocardial ischaemia. We propose that Hand1 is part of a novel regulatory pathway linking cardiac oxygen levels with oxygen consumption. Understanding hypoxia adaptation in the fetal heart may allow development of strategies to protect cardiomyocytes vulnerable to ischaemia, for example during cardiac ischaemia or surgery.

## Abstract 2

This study reveals a novel pathway that responds to hypoxia and modulates energy metabolism by cardiomyocytes in the mouse heart, thereby determining oxygen consumption.

## Author Summary

Regulation of oxygen usage in cardiomyocytes is of great medical interest, because adult cardiac tissue is extremely vulnerable to hypoxia during myocardial infarction and cardiac surgery. While some progress has been made toward protecting cardiomyocytes from hypoxia in these circumstances, it has been limited by a lack of understanding of endogenous oxygen-sensing pathways. In contrast to adult cardiac tissue, embryonic cardiomyocytes are highly resistant to hypoxia, although the mechanisms underlying this have hitherto been unclear. Using mice we show that the transcription factor Hand1 is expressed at high levels in the fetal heart, under direct control of HIF1α signaling, a pathway well known to respond to hypoxia. We show that Hand1 expression decreases at birth as the neonate is exposed to higher levels of oxygen. By experimentally increasing Hand1 expression in the neonatal heart, we see lower oxygen consumption in cardiomyocytes and this is caused by *Hand1* repressing key regulatory genes involved in cardiomyocyte lipid metabolism. This has the effect of decreasing mitochondrial ATP generation via the tricarboxylic acid cycle. Furthermore, we show that increasing Hand1 expression in adult transgenic hearts is protective against myocardial infarction, suggesting that a hypoxia–Hand1 pathway may also be of importance in the adult heart.

## Introduction

Adult cardiomyocytes are particularly vulnerable to hypoxia, which can result in cellular dysfunction and death. While some progress has been made towards protecting cardiomyocytes from the deleterious effects of low oxygen levels, cardiac ischaemia continues to result in high mortality and remains a clinical challenge [1]. Fetal cardiomyocytes, in contrast, are adapted to function at extremely low oxygen levels. In the relative hypoxia of the womb, the fetal heart generates ATP predominantly from glucose via glycolysis and energy generation shifts to mitochondrial β-oxidation of lipids and the tricarboxylic acid cycle as the primary source of energy for the heart only after birth, when oxygen levels are abundant [2],[3]. It is striking that to varying degrees this is reversed in the adult failing heart.

Little is known about the mechanisms regulating these changes in cardiac energy metabolism. Specifically, the molecular link between oxygen availability and molecular control of energy metabolism is currently ill-defined. In the mouse, cellular and metabolic changes in the heart occur rapidly after birth, the switch from glycolysis to β-oxidation of lipids and the tricarboxylic cycle occurring within a week of birth [2],[4]–[7]. Under conditions of “stress” such as those leading to cardiac hypertrophy and heart failure, fetal-type glycolytic energy metabolism is known to recommence [8]. This is accompanied in the failing heart by re-expression of several isoforms of enzymes, of transcription factors, and of structural and other proteins normally expressed in the fetal heart. Metabolic remodeling therefore appears to be part of a broader phenotypic switch between adult and fetal states [8]. Analyzing the mechanisms regulating changes in the heart during the transition from fetus to neonate, in particular the changing sensitivity to oxygen levels, may therefore inform efforts towards more effective therapeutic intervention for heart failure. Furthermore, understanding how the fetal heart is adapted to hypoxia may allow development of strategies to protect cardiomyocytes vulnerable to ischaemia, for example during myocardial ischaemic events or cardiac surgery [1].

We have previously reported studies suggesting a role for elevated levels of the cardiac transcription factor *Hand1* in the derangement of electrical conductivity and arrhythmia of the failing heart [9]. Here we show that cardiac levels of *Hand1* mRNA and protein fall immediately following birth and investigate the control and consequences of this postnatal down-regulation. Expression of *Hand1* mRNA is up-regulated by *HIF* signaling, and *Hand1* itself inhibits the expression of a number of genes involved in cardiomyocyte lipid metabolism. Through inhibition of lipid metabolism, *Hand1* reduces cellular oxygen consumption and cardiomyocyte vulnerability to ischaemia. We hypothesise that in the cardiomyocyte, *Hand1* is part of a pathway linking ambient oxygen levels to oxygen consumption through regulation of cellular lipid metabolism.

## Results

### Hand1 Expression Is Induced by Hypoxia Signaling in the Perinatal Heart

**Figure 1:**
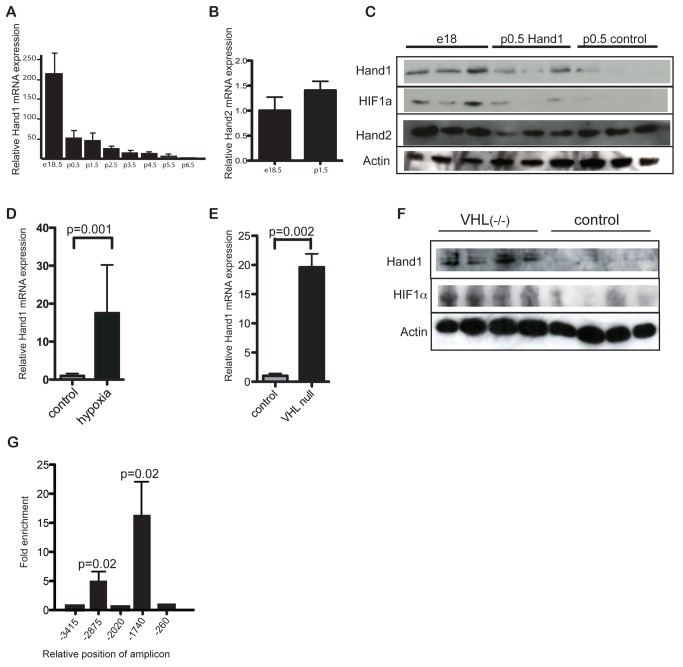
*Hand1* levels fall in the heart immediately following birth, under control of hypoxia signaling. (A) RTPCR for *Hand1* RNA from whole hearts of perinatal mice at a range of stages around birth, showing a steep decline in expression from birth. Levels expressed as a multiple of average 6-wk-old adult levels (*n* = 4 each group). (B) Levels of cardiac *Hand2* RNA do not fall at birth. Levels of *Hand2* at p1.5 normalised to e18.5 levels (*n* = 6 each group). (C) Western blot of protein extract from e18 (prenatal) control, p0.5 *XMLC-Hand1*, and p0.5 control hearts showing reduction in Hand1 but not Hand2 protein levels after birth, and persistence of Hand1 expression in *XMLC2-Hand1* hearts. (D) RTPCR showing increased *Hand1* RNA levels in hearts of adult wild-type mice incubated at 12% oxygen for 2 wk (“hypoxia”) over controls at normoxia (20% O_2_) (*n* = 4 each group) (*p* = 0.001, two-tailed *t* test). (E) RTPCR showing significantly increased *Hand1* RNA levels in the hearts of p0.5 neonatal *α-MHC-Cre::VHL^(fl/fl^* ^)^ mice compared with wild-type controls, *p* = 0.0002 two-tailed *t* test, *n* = 6 each group. (F) Western blot of protein extract from *VHL^(fl/fl)^* and control hearts at p0.5, showing elevation of Hand1 and HIF1α in *VHL^(fl/fl)^* hearts. (G) RTPCR of chromatin immunoprecipitation assay using anti-HIF1α antiserum and primers to the HIF motif-containing sequences in the *Hand1* promoter from e18 hearts, showing binding of HIF1α to two sites. Bars represent summation of three experiments, and results expressed as multiples of signal for nonamplified sequence. The *p* values are two-tailed *t* tests relative to nonamplified *γ-crystallin* primers.

*Hand1* mRNA is expressed at high levels in the developing embryonic heart [10],[11]. In contrast, only low levels have been reported in adult cardiac tissue [12]. We therefore investigated whether *Hand1* expression changed around birth or during subsequent growth and maturation of the heart. Using quantitative RTPCR analysis of cDNA from mouse hearts, we found that *Hand1* mRNA levels decrease rapidly in the immediate postnatal period (Figure 1A), whereas those of the related transcription factor *Hand2* remain largely unaltered (Figure 1B). We found that protein levels of Hand1 and 2 broadly follow those of mRNA (Figure 1C). In the early embryo, *Hand1* is expressed at high levels in the developing trophoblast and subsequently in the developing heart [11],[13]. Both tissues are known to be dependent on hypoxia signaling for normal development [14]–[17], and we therefore investigated whether the steep fall in perinatal cardiac Hand1 levels is due to the rapid change in ambient oxygen levels encountered by the neonate around birth.

We first tested whether ambient levels of oxygen affect *Hand1* levels in the adult mouse, incubating wild-type adult mice in 12% oxygen for 2 wk and comparing Hand1 expression levels with those of controls maintained under normoxic conditions. Such exposure to hypoxia resulted in 18-fold elevation of *Hand1* mRNA levels compared with controls (*n* = 4 each group, *p* = 0.001) (Figure 1D), indicating that cardiac Hand1 levels are directly or indirectly hypoxia-inducible.

We next tested whether neonatal *Hand1* expression is directly dependent on HIF1α signaling by examining *Hand1* mRNA levels in transgenic mice showing constitutive hypoxic signaling. Cardiac-specific deletion of *VHL* in *αMHC-cre::VHL^(fl/fl)^* neonates results in constitutive cardiac expression of *HIF1α* [18]. At p0.5, *Hand1* mRNA and protein were significantly elevated in *αMHC-cre::VHL* ^(fl/fl)^ hearts compared with nontransgenic controls (Figure 1E,F). Furthermore, chromatin immunoprecipitation (ChIP) showed binding of HIF1α to two of the five canonical HIF binding sites located in 5 kb of sequence upstream of the murine Hand1 transcriptional start site (Figure 1G).

### The Effects of Hand1 Misexpression in Neonatal Mouse Hearts

**Figure 2:**
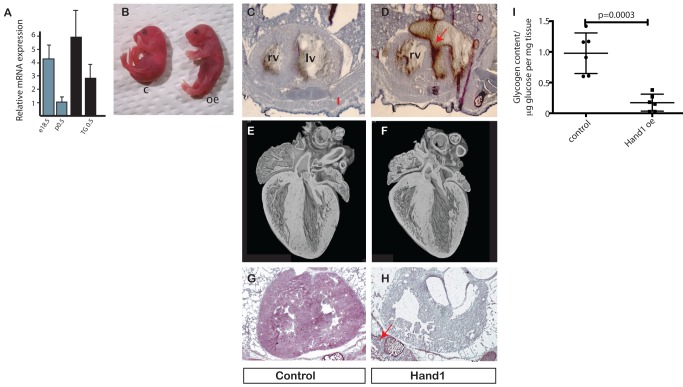
Prevention of neonatal *Hand1* down-regulation in transgenic mouse hearts leads to cardiomyopathy and death. (A) Cardiac RNA levels of *Hand1* in e18.5 and p0.5 wild-type, and e18.5 and p0.5 transgenic (TG 18.5 and TG0.5, respectively) showing RNA levels in the transgenic heart around 2.5 times that of wild-type p0.5 heart. (B) Cardiac *Hand1* elevating pups appear grossly normal, but are cyanosed (c, control; oe, Hand1 overexpressing). (C, D) H and E stain of cryostat section through thorax of control and *Hand1* overexpressing hearts, showing thin ventricular wall of the Hand1 overexpressing heart, and ventricular rupture (arrowed) with blood in the pericardial space (rv, right ventricle; lv, left ventricle). (E, F) EFIC sectioning and reconstruction of control and Hand1 overexpressing hearts from 4-h-old fostered pups, showing small size but no gross structural defect. (G, H) Periodic acid-Schiff stain of control and Hand1 overexpressing heart, showing decreased glycogen levels in *Hand1* overexpressing heart (purple). Glycogen stain in intercostal muscle of transgenic pup arrowed in (H). (I) Quantification of glucose enzymatically released from glycogen in hearts of neonates 2 h after caesarian section. Levels of glycogen in XMLC-Hand1 hearts are 17.5% of XMLC controls (*p* value, two-tailed *t* test, *n* = 6 hearts each group).

We then used the inducible *XMLC2-rtta::tet-Hand1* system (*XMLC2-Hand1*) [9] to determine the effect of prolonging *Hand1* expression in the neonatal heart. Inducing persistent *Hand1* transgene expression from the day before birth results in approximately 2.5-fold increase in *Hand1* mRNA, corresponding to 60% of fetal levels at p0.5 (Figure 2A). In subsequent studies, we examined the phenotype of male pups at p0.5, comparing induced *XMLC2-Hand1* pups with littermate controls expressing solely cardiac-specific *rTTA* transcription factor (*XMLC2-rTTA*).

Prior to birth, *XMLC2-Hand1* mice are present in expected Mendelian ratios (14 *XMLC2-Hand1* out of a total of 30 pups recovered at e18 after 48 h of maternal doxycycline induction, expected *n* = 15). The 3D reconstruction of high-resolution episcopic datasets [19],[20] revealed no significant structural difference between *XMLC2-Hand1* and control littermates (Figure S1A). *XMLC2-Hand1* pups exhibit respiratory distress shortly after birth, whereas control littermates appear normal (Figure 2B and Movie S1). *XMLC2-Hand1* hearts were significantly lighter than controls (10.1±0.62 mg versus 7.08±0.55 mg; two-tailed *t* test *p* = 0.007, *n* = 6 each group). Genotype analysis indicated that Hand1 up-regulation also led to high rates of neonatal death in the immediate neonatal period (six *Hand1* up-regulating pups out of 56 expected from 12 litters, collected at p0.5) with left ventricular cardiac rupture detected in two out of the three dead neonates collected (Figure 2C,D). As neonatal death rates in *XMLC2-Hand1* are so high, we performed caesarian section on gravid *XMLC2-rTTA* female mice pregnant with *XMLC2-Hand1* and control pups. Neonates were fostered to wild-type females, and at 4 h *postpartum*, all pups were sacrificed and genotyped. Sectioning/reconstruction revealed that *XMLC2-Hand1* hearts were smaller than controls, but with no gross anatomical derangement (Figure 2E and F and Figure S1B).

Although histological analysis revealed no obvious tissue derangements or increase in apoptosis in *XMLC2-Hand1* mice compared with controls (Figure S1), PAS staining revealed a marked depletion of glycogen in the myocardium of Hand1-upregulating neonates, consistent with exhaustion of glycolytic substrate (Figure 2G,H). We went on to analyse glycogen levels in the hearts of *XMLC2-Hand1* mice compared with controls. We found that glycogen levels in *XMLC2-Hand1* mice 2 h after caesarian section were reduced compared with *XMLC2-rTTA* controls (0.17±0.056 versus 0.98±0.153 mmol glygogen-derived glucose per mg heart tissue, *n* = 6, *p* = 0.0003, two-tailed *t* test) (Figure 2I).

### Transcription Changes in Hand1 Up-Regulating Hearts

Glycogen depletion suggested significant changes in neonatal myocardial energy metabolism as a result of *Hand1* up-regulation, and we therefore performed Affymetrix microarray analysis to monitor changes in the myocardial transcriptome (Table S1). Gene ontology analysis revealed overrepresentation of genes involved in fatty acid metabolism amongst those showing significant changes as a result of elevated Hand1 expression (Table S2).

**Figure 3:**
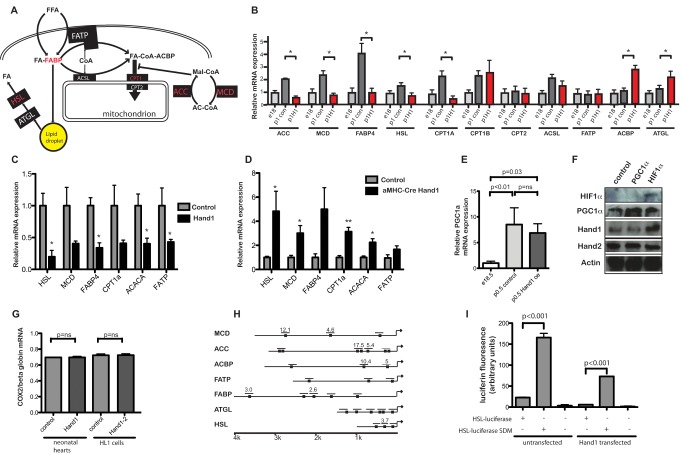
Prolongation of neonatal cardiac *Hand1* expression prevents transcriptional up-regulation of lipid metabolizing genes. (A) Schematic showing myocardial lipid metabolism (adapted from Kodde et al. [55]). (B) RTPCR showing RNA expression in e16 control, p0.5 control, and p0.5 hand1 up-regulating hearts. Levels of *ACC*, *MCD*, *FABP4*, *ACSL*, *CPT1A CPT1B*, and *HSL* are significantly up-regulated around birth (*p*<0.05 two-tailed *t* test, *n* = 4 each group). No postnatal rise in *ACC*, *MCD*, *FABP4*, *CPT1A*, and *HSL* is seen in Hand1 up-regulating hearts. Significantly increased RNA expression of *ACBP* and *ATGL* is seen in Hand1 up-regulating hearts. Genes whose expression is reduced in Hand1 overexpressing hearts are in red in (A). *ACC*, acetyl coA carboxylase; *MCD*, malonyl coA decarboxylase; *FABP*, fatty acid binding protein; *FATP*, fatty acid transport protein; *ACSL*, acyl coA synthase long chain 1; *HSL*, hormone sensitive lipase; *ATGL*, adipose triglyceride lipase; *ACBP*, acylcoA binding protein; *CPT*, Carnitine Palmitoyl Transferase. (C) RTPCR of mRNA from 2-mo-old adult XMLC2-Hand1 mice following doxycycline induction for 2 wk, showing changes in expression of RNA encoding fatty acid metabolising proteins relative to control non-up-regulating mice (*p<0.05, **p<0.005, two-tailed *t* test, *n* = 4 each group). (D) RTPCR of mRNA from e14.5 embryo hearts from αMHC-Cre::Hand1^(fl/fl)^ and control pups, showing up-regulation of genes encoding fatty acid metabolising enzymes(*p<0.05, two-tailed *t* test, *n* = 4 each group). (E) RTPCR showing significant *PGC1-α* elevation in the heart around birth (*n* = 4 each group, *p* = 0.008, two-tailed *t* test), with no significant drop in *Hand1* up-regulating hearts (*p* = 0.26, *n* = 6 each group). (F) Western blot of protein extract from cultured HL1 cardiomyocytes nontransfected (“control”) and transfected with *PGC1-α* and *HIF1*, showing no elevation of Hand1 in *PGC1-α* elevated PGC1*α* and Hand1 but not Hand2 protein expression in HIF1 expressing cells. (G) PCR of nuclear genomic (*globin*) and mitochondrial (*COX2*) DNA showing unchanged ratio in *Hand1* elevating neonatal hearts and Hand1-transfected HL1 cells compared with controls, implying no change in mitochondrial number. Control HL1 cells are transfected with an empty vector. (H) Map of 5′ promoters of several putative *Hand1* transcriptional targets in the e18.5 heart. Numbers refer to fold enrichment over *γ crystallin* in chromatin immunoprecipitation assay using anti-Hand1 serum. For more detailed chromatin immunoprecipitation data, please see Text S1 and Figure S2. (I) Site-directed mutagenesis of the Hand1-binding canonical CANNTG e-box in the 5′ HSL luciferase promoter de-represses expression of luciferase in HL1 cells, both in untransfected cells and cells stably expressing Hand1 (transfections in triplicate, measurement in quadruplicate, *p* values, two-tailed *t* test).

Expression of several genes encoding enzymes involved in cardiac fatty acid metabolism is up-regulated at birth. Using RT-PCR, we found that *XMLC2-Hand1* hearts failed to up-regulate a subset of these genes. We found that at birth, expression of several genes encoding enzymes involved in lipid and acylcarnitine metabolism are up-regulated in the wild-type heart at p0.5, including *ACC* (acetyl coA carboxylase), *MCD* (malonyl coA decarboxylase), and *CPT* isoforms (carnitine palmitoyl transferase) as previously reported [21]–[24], along with *FABP* (fatty acid binding protein), *FATP* (fatty acid transport protein), *ACSL* (acyl coA synthase long chain 1), *HSL* (hormone sensitive lipase), and *ATGL* (adipose triglyceride lipase) (Figure 3A,B). Prolongation of Hand1 expression prevented the postnatal increase in expression of *MCD*, *ACC*, *FABP*, *HSL*, and *CPT1a* (but not *CPT1b* or *CPT2*). We found no significant differences in expression of mRNA encoding PPAR isoforms (Figure S2), glycolytic genes, or mitochondrial electron transport complexes between Hand1 up-regulating and control neonatal hearts (Figure S2). We detected broadly similar changes in gene expression following up-regulation of Hand1 in adult transgenic XMLC2-Hand1 mice inducibly up-regulating Hand1 (Figure 3C) and in stably transfected HL1 lines (Figure S2). Reduction of Hand1 expression levels in siRNA stably transfected HL1 cells, and e14.5 αMHC-Cre::Hand1^(fl/fl)^ embryos led to a general increase in expression of lipid metabolising genes (i.e., opposite to changes in Hand1 up-regulating cells) (Figure 3D and Figure S2).

Previously, it has been found that an elevation in *PGC1-α* expression drives cardiac mitochondrial biogenesis, as part of postnatal cardiac energetic remodeling [25]. We confirmed that levels of *PGC1*-α mRNA rise in the postnatal mouse heart, but there was no significant change in *PGC1-α* mRNA levels in *Hand1* up-regulating hearts (Figure 3E). We found no elevation of *Hand1* protein expression in HL1 cells transfected with *PGC1-α* (Figure 3F). Furthermore, we detected no difference in mitochondrial/nuclear DNA ratio in *XMLC2-Hand1* hearts compared with controls, or in *Hand1* transfected HL1 cell lines compared with lines stably expressing shRNA against *Hand1* (Figure 3G and Figure S2), nor of genes expressing mitochondrial respiratory complex isoforms (Figure S2). We detected no significant change in expression of mRNA encoding either *PGC1-α* or its downstream target gene *ERR-α* in hearts from αMHC-cre::VHL^(−/−)^ neonates (Figure S2). These data imply that *Hand1* in the neonatal heart acts independently of *PGC1-α*.

We next carried out ChIP, assaying canonical E-boxes (CAnnTG) in the 5′ promoters of the *ACC*, *MCD*, *ACBP*, *FABP4*, *FATP*, *HSL*, and *ATGL* genes, using a Hand1 antibody (Figure 3H and Figure S2). This revealed that Hand1 binds to several (but not all) E-boxes in the 5′ promoters of *ACAC*, *MCD*, *FABP4*, *ACBP*, and *HSL* promoters in vivo (albeit with varying degrees of avidity). No binding of Hand1 to these sites was detectable in chromatin isolated from e14.5 *Hand1* null hearts (*αMHC-Hand1^(fl/fl)^*) (Figure S2) [26]. This suggests that repression by Hand1, at least in part, reflects direct control of these genes.

We then went on to confirm that Hand1 directly regulates one of these putative target genes. We cloned a 1 kb fragment of the mouse HSL promoter and ligated it into the pGL3 luciferase vector (Promega), and performed site-directed mutagenesis to abolish the middle e-box site, which we found bound Hand1 protein in the chIP assay (Figure 3I). Mutation of this e-box site led to a 7-fold increase in luciferase expression over the e-box containing promoter in untransfected HL1 cells, and a 13-fold increase in HL1 cells stably transfected with Hand1. Lower basal levels of promoter activation were found in Hand1 expressing cells (Figure 3I).

### Prolonged Perinatal Expression of Cardiac *Hand1* Inhibits Myocardial Lipid Metabolism

**Figure 4:**
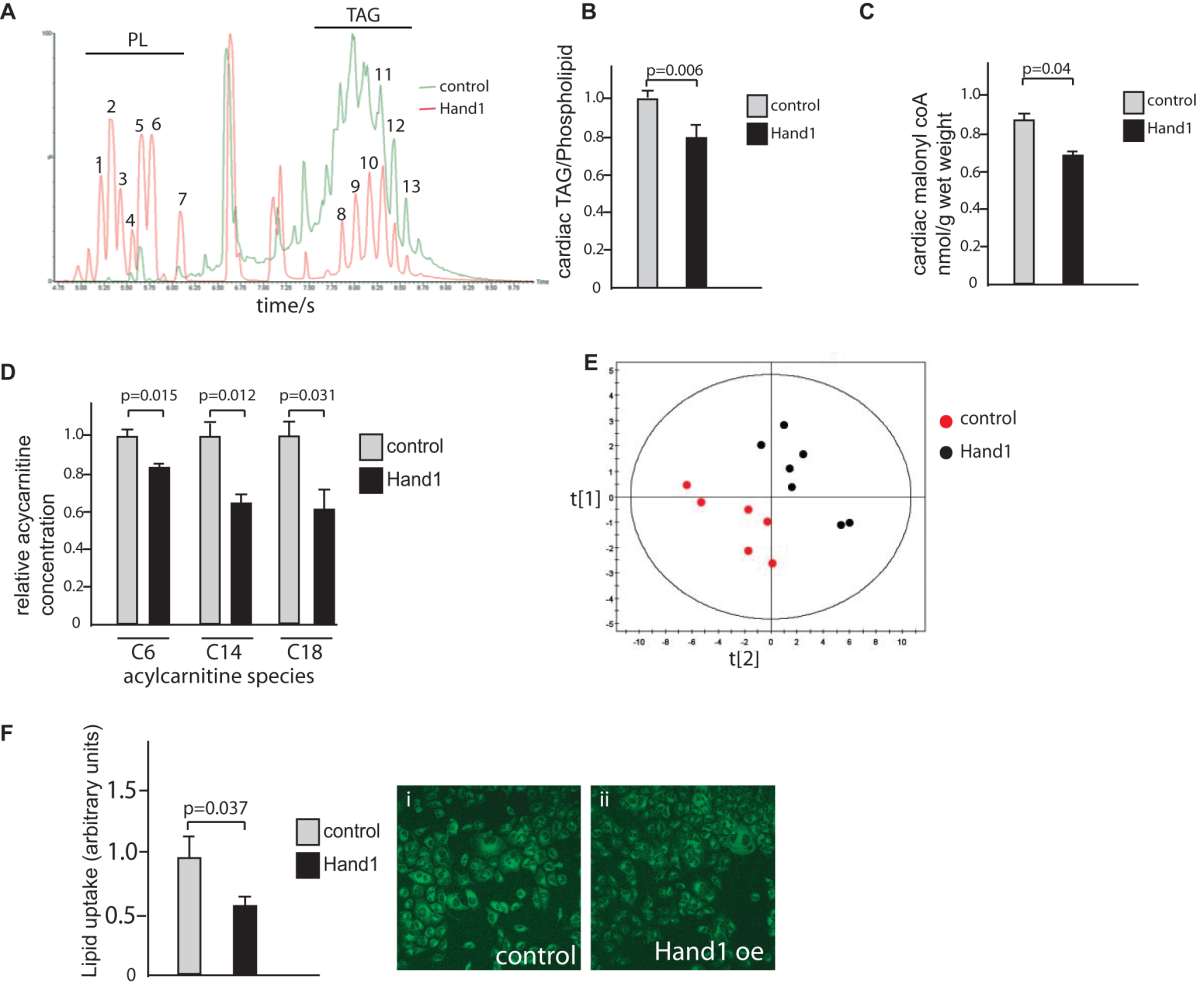
Lipid metabolism is inhibited in neonatal *Hand1* overexpressing hearts. (A) LC-MS trace showing typical output for intact lipid extracted from control hearts (green trace) and Hand1 up-regulating hearts (red), showing significantly lower levels of triacylglycerides (TAG) compared to phospholipid (PL) in Hand1 up-regulating hearts. (B) Quantitative analysis of cardiac triacylglyceride levels showing significant reduction in Hand1 up-regulating hearts, expressed as the ratio of TAG to phospholipid (*n* = 6 each group, *p* = 0.006, two-tailed *t* test). (C) Quantitative analysis of cardiac malonyl coA levels showing significant reduction in Hand1 up-regulating hearts (*n* = 6 each group, *p* = 0.04, two-tailed *t* test). (D) Reduced levels of C6, C14, and C18 containing acylcarnitine species in Hand1 prolonging neonatal hearts compared with controls. For full dataset, please refer to Table S3. (E) Multivariate partial least squares discriminant analysis (PLS-DA) scores of acylcarnitine profiles showing a significant decrease in global levels of acylcarnitines in Hand1 up-regulated hearts relative to controls (R^2^X = 33%, R^2^Y = 62%, and Q^2^ = 48%). (F) BODIPY-500/510C_1_, C_12_ uptake is significantly reduced in HL1 cells by transfection with Hand1. Graph shows quantification of fluorescence in Hand1 transfected and nontransfected controls, 10 high power fields each. (i) and (ii) show representative fluorescence micrographs of control and Hand1 transfected cells following labeled lipid incubation (*p* = 0.037, two-tailed *t* test).

We examined whether Hand1 has an effect on lipid metabolism, as suggested by our transcriptomic analysis. We found lower overall levels of triacylglycerides and reduced levels of malonyl CoA in neonatal *Hand1*-persisting hearts compared with controls (Figure 4A–C). Multivariate analysis confirmed an overall decrease in lipid incorporation into acylcarnitine metabolites, with significantly decreased levels detected of C6-, C14-, and C18-containing acylcarnitine species (Figure 4D,E and Table S3). We also found that uptake of the fluorescently labeled lipid substrate BODIPY-500/510C_1_, C_12_ is reduced by 37.1% in Hand1 transfected cells compared with controls (*p* = 0.034 two-tailed *t* test) (Figure 4F). Therefore, elevated *Hand1* expression levels not only decreases incorporation of lipid into cellular metabolic processes via targeting expression of several enzymes of lipid/acylcarnitine metabolism, but also by cardiomyocyte lipid uptake.

### Hand1 Reduces Cellular Oxygen Consumption

**Figure 5:**
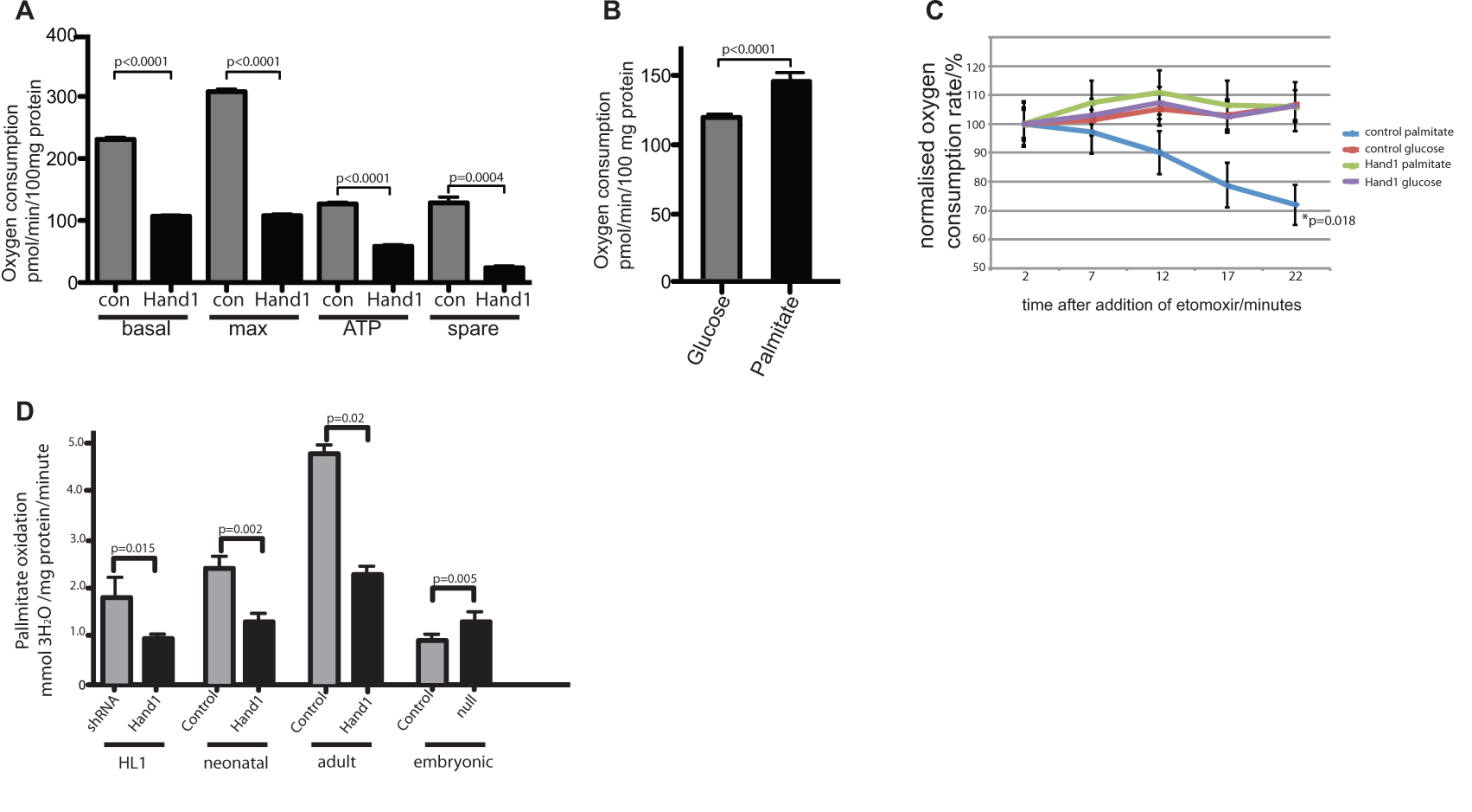
*Hand1* reduces oxygen consumption and lipid oxidation in stably transfected HL1 cells and primary cardiomyocytes. (A) In HL1 cells stably transfected with *Hand1*, basal respiration (basal) maximal respiratory capacity (max), ATP production (ATP), and spare respiratory capacity (spare) are all significantly reduced compared to empty-vector transfected controls, using medium that contains 5.5 mmol glucose and 2 mmol pyruvate (five duplicate wells for each measurement, experiments repeated three times, two-tailed *t* test). (B) Oxygen consumption is increased in HL1 cells following incubation with 20 mmol palmitate for 20 min compared with cells incubated in 12 mmol glucose (15 replicate wells, two-tailed *t* test). Substrate was added to basal incubation medium that contains 5.5 mmol glucose and 2 mmol pyruvate. (C) The CPT1 inhibitor etomoxir (1 µM) reduces oxygen consumption in nontransfected but not *Hand1* expressing HL1 cells when incubated with 20 mM palmitate (*p* = 0.018 AUC ANOVA, 15 wells per sample). No reduction in oxygen consumption is seen in either cell type when cells incubated with 12 mM glucose are treated with etomoxir (i.e., undergoing glycolyic respiration). Measurements are carried out after treatment with oligomycin and FCCP. (D) Hand1 reduces palmitate oxidation in cardiomyocytes. HL1 cells stably expressing *Hand1* generate significantly less ^3^H_2_0 from ^3^H labeled palmitate compared with a line stably expressing an shRNA construct directed against *Hand1*, showing lower levels of lipid oxidation. Bars represent sum of three experiments (*p* = 0.015, two-tailed *t* test). Primary cultures of Hand1 up-regulating neonatal cardiomyocytes (four hearts each group) and adult hearts (two hearts each group) generate significantly less ^3^H_2_0 than controls (two-tailed *t* test). Primary cultured cardiomyocytes from e15 Hand1null αMHC-Cre::Hand1^(fl/fl)^ (three hearts each group) exhibit significantly increased levels of lipid uptake (two-tailed *t* test).

To test the hypothesis that the reduction in cardiomyocyte lipid uptake and synthesis mediated by *Hand1* will be reflected in decreased cellular oxygen consumption, we tested the effect of *Hand1* expression on oxygen consumption of stably transfected HL1 cardiomyocyte lines. We found basal oxygen consumption reduced by 53.7% (*p*<0.0001 two-tailed *t* test), maximal respiratory capacity by 64.9% (*p*<0.0001), ATP production by 54.7% (*p*<0.0001), and spare respiratory capacity (a measure of unused respiratory capacity) by 82.1% (*p* = 0.0004) compared with controls, transfected with an empty vector (empty vector control used throughout) (Figure 5A). Incubation of HL1 cells with excess of palmitate or glucose has the effect of driving the cell towards either β-oxidation of lipid or glycolysis, respectively. Lipid oxidation is reflected by a significant increase in oxygen consumption (Figure 5B). A total of 1 µM of the *CPT1* inhibitor etomoxir blocks the import of fatty acids to the mitochondrion [27] and abolishes this increase in oxygen consumption in nontransfected palmitate-treated control cells (Figure 5C). However, oxygen consumption in *Hand1* transfected cells incubated with palmitate does not change in response to etomoxir, implying that no lipid oxidation is occurring in *Hand1* transfected cells (Figure 5C). We went on to assay the rate of Palmitate oxidation directly in *Hand1* transfected and control cells transfected with an shRNA construct directed against *Hand1*, by incubating cells in ^3^H-labelled palmitic acid and measuring generated ^3^H_2_O [28]. We found a reduction of 51% in the rate of generation of ^3^H_2_O in cells stably transfected with Hand1 compared with Hand1 shRNA transfected cells (Figure 5D). We performed ^3^H-labelled palmitic acid oxidation studies in primary cardiomyocyte cultures from XMLC2-Hand1 neonates and adults, and found that elevation of Hand1 expression also leads to a significant reduction in palmitate oxidation in these cell types. Furthermore, cardiomyocytes from e15 Hand1null αMHC-Cre::Hand1^(fl/fl)^ exhibit significantly increased levels of lipid uptake compared with αMHC-Cre::Hand1^(+/+)^ littermates (Figure 5D). Taken together, these results show that *Hand1* reduces oxygen consumption in HL1 cardiomyocytes, by inhibiting mitochondrial β-oxidation of fatty acids.

### Hand1 Controls Mitochondrial Function

In order to investigate the effect of Hand1 levels on myocardial energy generation, we went on to examine mitochondrial function. We examined p.05 neonatal hearts from XMLC2-Hand1 and control pups.

**Figure 6:**
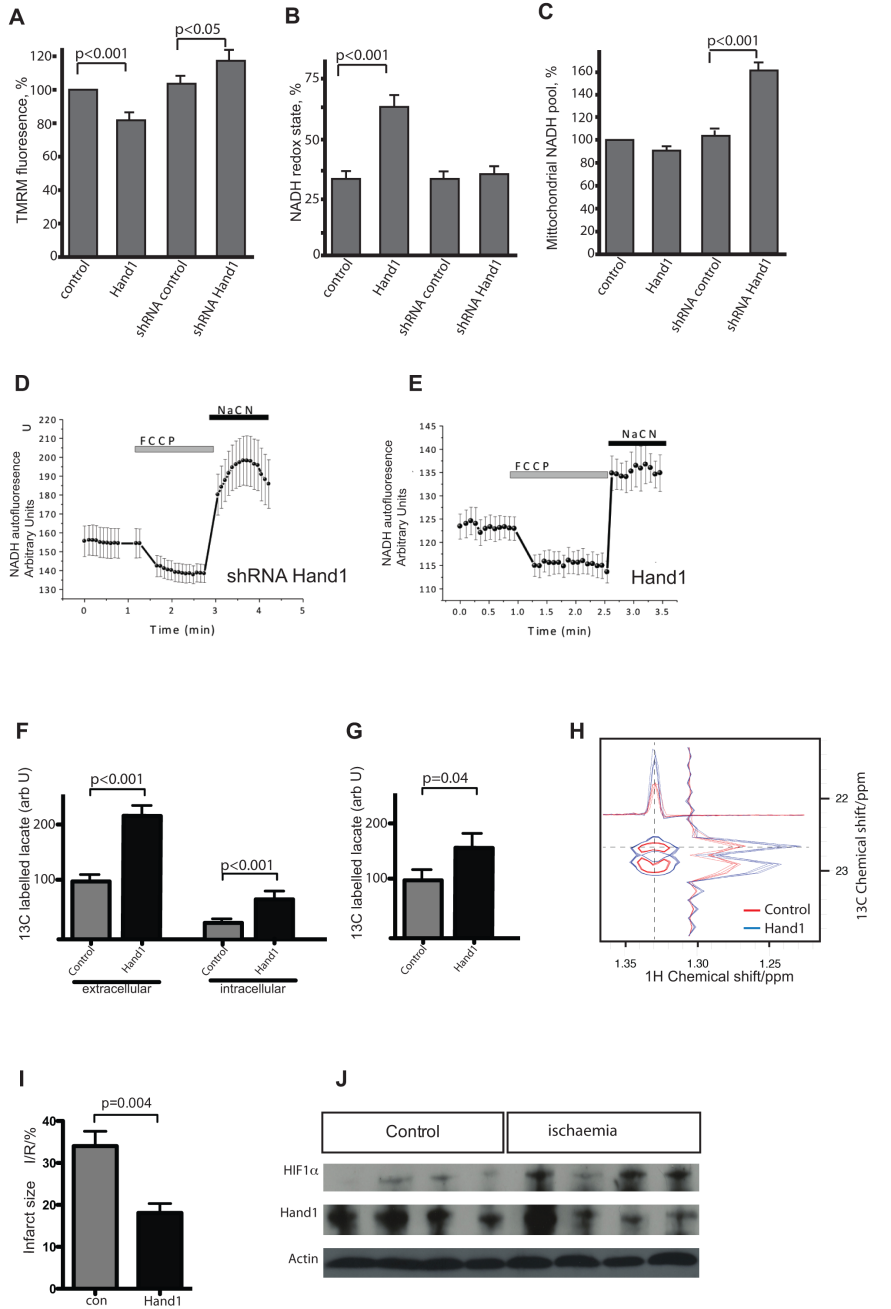
Hand1 levels control mitochondrial function, metabolic flux, and ischaemia susceptibility in cardiomyocytes. (A) TMRM fluorescence measured in stably transfected HL1 cell lines, showing significantly reduced mitochondrial inner membrane potential in Hand1 up-regulation, and significantly increased potential in shRNA-expressing lines knocking down Hand1 expression (number of cells analysed = 26, 34, 23, and 23, respectively) (*p* = two-tailed *t* test). (B) Significantly increased NADH redox state in Hand1 expressing stable HL1 lines, measured by NADH autofluoresence. (C) Hand1 up-regulating stably transfected HL1 cells display a significantly reduced mitochondrial NADH pool compared with controls, whereas knockdown of Hand1 results in an increase in the NADH pool, estimated as a difference in fluorescence (arbitrary U) between responses to FCCP and NaCN. (D) Example of an NADH autofluoresence trace of a stably transfected Hand1 shRNA HL1 cell, showing maximally oxidized state in response to the uncoupler FCCP, and minimally oxidized state in response to sodium cyanide, in comparison to the trace for Hand1 transfected cell. (E) A typical NADH autofluoresence trace of a stably transfected Hand1 line, showing much lower NADH levels, as defined by the ratio of maximally and minimally oxidized states. (F) ^13^C labeling of lactate is increased in supernatant (extracellular) and cell extract (intracellular) of stably transfected Hand1 up-regulating HL1 cells after labeling of cells with uniformly labeled ^13^C glucose (*n* = 4 for each measurement), measured by ^1^H. (G) Increased detection of ^13^C labeling of lactate in adult XMLC-Hand1 mice 1 h after administration of uniformly labeled ^13^C glucose (*n* = 6 each group, *p* = 0.04, two-tailed *t* test). (H) ^1^H NMR showing increased ^13^C incorporation into lactate from [U ^13^C_6_]-glucose in Hand1 up-regulating HL1 cells (blue trace) compared to controls (red trace). (I) Following Langendorff perfusion and 35 min of global ischaemia followed by 30 min of reperfusion, hearts from 2-mo-old *Hand1* up-regulating *XMLCrTTA::tetHand1* adult mice following 1 mo of doxycycline induction exhibit a 47% reduction in infarct size, infarcted tissue area expressed as a proportion of total at-risk tissue area (I/R) (*n* = 6, *p* = 0.004, two-tailed *t* test). (J) Western blot of protein extracts of wild-type adult Langendorff perfused hearts following 30 min of global ischaemia. Increased levels of HIF1α are detected following global ischaemia, but no difference in Hand1 protein levels is apparent.

Mitochondrial membrane potential (Δψ_m_) is an indicator of mitochondrial energetic state. HL1 cells stably transfected with Hand1 demonstrate significant reduction in Δψ_m_, assayed by TMRM fluorescence analysis, to 81.4%±5.2% of controls (*n* = 26 cells; *p*<0.001; Figure 6A). However, Hl1 cells stably transfected with an shRNA construct down-regulating Hand1 showed a significantly increased Δψ_m_ (to 116.8%±8% of control; *n* = 23; *p*<0.05; Figure 6A). This implies lower mitochondrial ATP generation in Hand1 up-regulating HL1 cells.

The redox state of mitochondrial NADH is a function of respiratory chain activity and substrate turnover. We measured the resting level of NADH autofluorescence in HL1 cells, which was then expressed as the “redox index,” a ratio of the maximally oxidized and maximally reduced signals [29]. The dynamic range of the signals was defined by obtaining the maximally oxidized signal following the response to 1 µM FCCP (which stimulates maximal respiration and fully oxidises the mitochondrial NADH pool). In these conditions, mitochondrial NADH is taken as fully oxidised and defined as 0%. The maximally reduced signal was then defined as the response to 1 mM NaCN (which fully inhibits respiration), preventing NADH oxidation, and so promoting maximal mitochondrial NADH reduction. In these conditions, NADH is taken as 100% reduced. HL1 cells down-regulating Hand1 did not significantly change the NADH redox state (35.7%±2.89%; *n* = 23 compare to 32.6%±2.4% in control shRNA transfected cells). In contrast, Hand1 up-regulation significantly increased NADH redox state (64.9%±5.9%, *n* = 25; *p*<0.001; Figure 6B), suggesting inhibition of mitochondrial respiration. It should be noted that Hand1 down-regulation and Hand1 up-regulation had an opposite effect on the total mitochondrial pool of NADH (Figure 6C). We found that Hand1 down-regulation significantly increases the NADH substrate pool (to 156%±6.5% of control; *p*<0.001) and Hand1 up-regulation significantly decreases the NADH pool in mitochondria (87.5%±4.6% of control; *p*<0.05).

We then carried out glucose flux analysis on stably transfected HL1 cell lines, using uniformly labeled ^13^C-Glucose, incubating for 4 h and analysing ^13^C lactate levels with ^1^H NMR. We found significant elevation of lactate production in Hand1 overexpressing cells compared with control Hand1 shRNA down-regulating cells (Figure 6F,H), and significantly elevated ^13^C labeling of lactate in adult XMLC-Hand1 hearts compared with controls (Figure 6G). We also found that primary cultured cardiomyocytes from Hand1 up-regulating neonatal hearts acidified the extracellular medium in a seahorse XF assay faster than control cardiomyocytes (Figure S3), implying that these cells are more glycolytic.

Taken together, these results imply that the effect of Hand1 is to reduce mitochondrial energy generation, and to switch cellular metabolism from aerobic glycolysis and mitochondrial energy generation to anaerobic glycolysis.

### Modulation of Hand1 Expression in a Mouse Model of Cardiac Ischaemia

Since up-regulation of *Hand1* reduces cardiomyocyte oxygen consumption, we hypothesised that an increase in cardiac *Hand1* levels may enhance tolerance to ischaemia. We therefore tested the effect of Hand1 up-regulation in an animal model of myocardial ischaemia. Hearts were removed from adult (2 mo old) up-regulating (*XMLC2-Hand1*) and control (*XMLC2-rTTA*) mice after 1 mo of doxycycline administration, and subjected to 35 min of global ischaemia during Langendorff perfusion, followed by 30 min of reperfusion and infusion with prewarmed triphenyltetrazolium chloride as previously described [30]. Overexpression of the Hand1 transgene resulted in a 47% reduction in tissue death compared with control mice (*p* = 0.004) (Figure 6I) consistent with elevated *Hand1* levels reducing cardiomyocyte oxygen consumption. Following 30 min of global ischaemia of Langendorff-perfused adult wild-type hearts, we were able to detect increased levels of HIF1α protein, but not Hand1 protein, using Western blotting (Figure 6J). This implies that Hand1 is not involved in the acute response to hypoxia/ischaemia.

We have previously found that adult *Hand1* up-regulating hearts display a heart-failure-like phenotype of low threshold for ventricular arrhythmia and a diastolic defect [9]. As energy generation is also remodeled in failing hearts [31],[32], we measured PCr/Cr in Hand1 up-regulating adult mouse hearts. We found significant reductions in PCr/CR ratio (1.25±0.25 for controls versus 0.88±0.23 in Hand1 overexpression; *p* = 0.037) and PCr/ATP (1.70±0.06 versus 1.37±0.126; *p* = 0.0425) in *Hand1* up-regulating hearts (Table S4). Therefore, ischaemia protection in *Hand1* up-regulating hearts occurs at the expense of ATP production—that is, the same strategy employed in the fetal heart.

## Discussion

The mammalian cardiomyocyte is exposed to a large range of oxygen concentrations during development and terrestrial life. The ability of the cardiomyocyte to function in extremely low levels of oxygen is lost after birth, with serious medical consequences in the ageing human. Here we propose that the transcription factor *Hand1* is part of a novel metabolic pathway adapting the embryonic heart to varying levels of hypoxia during development, birth, and adulthood. This pathway may be of significance in the adult during heart failure, as Hand1 is one of the “fetal” genes up-regulated in the failing cardiomyocyte. The links between heart development and heart failure are becoming more apparent, and may provide clues to future therapies.

There is circumstantial evidence connecting *Hand1* expression with hypoxia during development. Hypoxia inducible factor (*HIF*) signaling is essential for formation of the placental trophoblast [14],[15], embryonic heart [16],[17], and developing nervous system [33]. Strikingly, these areas overlap *Hand1* function in the developing embryo [10],[11]. Interestingly, *HIF* signaling is thought to be activated in the failing heart [34], where *Hand1* is up-regulated [9]. Therefore, our data fit with a model whereby Hand1 expression is under control of hypoxia signaling in both the fetal and adult heart. Cardiac overexpression studies of the type described in this report are unable at present to differentiate between effects on the left and right ventricle. Development of reliable chamber-specific transgene expression is awaited for these studies. We found that cardiac-specific Hand1 null hearts exhibit up-regulation of the genes encoding proteins involved in lipid metabolism that are down-regulated in Hand1 overexpressing neonates. We also found that lipid oxidation in these hearts is increased relative to controls. These mice die *in utero* around e16–17 (our unpublished data and Mcfadyen et al. [26]). It is possible that the cause of death is an increase in oxygen demand due to up-regulated lipid metabolism. More broadly, the contribution of metabolic regulation to control of normal embryonic development is not yet clear.

The key adaptation of the fetal heart to hypoxia is the generation of ATP from oxygen-sparing glycolysis rather than oxygen-expensive lipid oxidation [35], although the absolute rates of glucose oxidation versus glycolysis are as yet unclear. There are several lines of evidence suggesting that selection of energetic substrate is coupled to ambient oxygen levels, and regulation of cellular lipid oxidation is a key mechanism to determine oxygen consumption. Experimental inhibition of cellular lipid metabolism by etomoxir, a *CPT1* antagonist that prevents mitochondrial long-chain fatty acid import, shifts energy metabolism to glycolysis, leading to lower myocardial oxygen consumption, and protects against myocardial ischaemia [36],[37]. Exposure of neonatal rats to hypoxia results in a decrease in overall lipid content and remodeled acyl-carnitine metabolism, resembling the effect of persistent Hand1 expression on lipid metabolism [38].

Our understanding of the molecular mechanisms linking lipid oxidation rates with ambient oxygen in the heart, specifically around birth, is incomplete. It is known that *PGC1-α* has a role in postnatal maturation of cardiac metabolism via regulation of mitochondrial number [25]. The effect of *Hand1* on the postnatal heart seems to be independent of *PGC1-α*. This is supported by the finding that overexpression of *PGC1-α* has a limited effect in the adult mouse heart [39] in contrast to the effects of *Hand1* overexpression [9]. The transcriptional control of *PGC1-α* remains mysterious. We found that PGC1-α mRNA levels were not altered in neonatal VHL null hearts (Figure S2), implying that expression of this gene is not affected by changes in cardiac HIF signaling levels at birth.

Our data show that the effect of Hand1 in the heart is to down-regulate mitochondrial metabolism as well as lipid metabolism, reflected by changes in mitochondrial morphology, membrane potential, and glucose flux. This mechanism leads to an additional layer of regulatory complexity, as several glycolytic enzymes are known to be directly regulated by HIF signaling [40]. HIF signaling is thus likely to regulate several metabolic pathways in the neonatal heart in parallel. This may lead to an increased degree of metabolic flexibility, as evidenced by the fact that the phenotype of cardiac Hand1 up-regulating neonates is more severe than that of αMHC-Cre::VHL^(fl/fl)^ neonates, which are born with relatively normal cardiac morphology and die with cardiac arrhythmia by the second postnatal week [41].

The idea that the same pathways are active in the fetus and adult heart is attractive, as it goes some way towards explaining the basis of the re-expression of the “fetal gene expression programme” seen in heart failure [8]. Perhaps a more accurate term is “hypoxia adaptive gene expression programme.” This is adaptation of the cardiomyocyte to low oxygen via metabolic and contractile gene isoform expression switching, and occurs to preserve oxygen at the expense of ATP production and lower cardiac output, as occurs in persistently Hand1 expressing neonatal hearts. There is evidence that this occurs in healthy humans. Healthy volunteers have been shown to up-regulate cardiac glucose oxidation at altitude [42], and it was recently found that healthy, young volunteers suffered what is essentially a reversible cardiomyopathy involving decreased ATP production and diastolic dysfunction on ascent of Everest [43]. Indeed, protection of the mouse heart against ischaemia by etomoxir occurs at the expense of ATP production and decreased lipid oxidation [37]. Remodeling of energy metabolism may be adaptive in the short term to protect against hypoxia, but is associated with a poor long-term clinical outcome in human heart failure [31],[32]. Our data showing that Hand1 is not significantly induced by ischaemia in the Langendorff perfused heart suggest that a hypoxia–Hand1 pathway is not involved in the response to acute ischaemia. This pathway seems more likely to be important in the response of the myocardium to chronic or repeated hypoxia/ischaemia. Interestingly, “hibernating” myocardium, whose function is temporarily decreased by repeated hypoxia, has been shown to revert to glycolysis [44],[45].

Our Langendorff perfusion data suggest that the HIF1α/Hand1 pathway may be active in the adult heart. However, this must be regarded as preliminary evidence at the moment. While this assay has proved to be a robust, reproducible assay for ischaemia/reperfusion studies, there are some important caveats when extrapolating Langendorff data to whole-animal physiology. The fact that the heart is removed from the mouse and perfused is clearly a major factor in this. Furthermore, the perfusate substrates contained in the Krebs-Henseleit buffer used in this assay are predominantly crystalloid, and are designed to optimize performance of the isolated heart, rather than to recapitulate physiological conditions [46]. The supraphysiological glucose concentrations in the Langendorff perfusate would, in theory, push the hearts towards a more glycolytic metabolism, potentially leading to an underestimate of the effects of Hand1 with respect to ischaemia protection. While it is theoretically possible to gain some measure of cardiac contractile function with a ventricular balloon in the Langendorff assay, we did not measure “cardiac function” in our Langendorff assay. We have argued in the past that such measurements in the Langendorff system are prone to artifact [46]. *In vivo* models of myocardial infarction will be necessary to investigate formally a role for modulating Hand1 levels in myocardial ischaemia protection. However, we have previously shown that adult Hand1 up-regulating mouse hearts display a diastolic defect without significant systolic dysfunction at steady state [9]. Studies are now ongoing to investigate formally a potential role for Hand1 in myocardial infarction.

Finally, our finding that Hand1 activity forms part of the regulatory mechanism adapting the fetal heart to intrauterine hypoxia may have clinical relevance. There is a growing body of evidence suggesting that cardiomyocyte lipid metabolism is of importance in determining oxygen consumption and therefore susceptibility to ischaemia [37],[47],[48]. It has also been shown that tight control of lipid metabolism is important in modulating oxygen consumption; overexpressing VLDL receptors in transgenic mouse hearts increases mortality following experimental myocardial infarction, presumably by increasing oxygen consumption via increasing lipid substrate presentation to the cardiac mitochondria [49]. Our data on the cardioprotective effects of Hand1 expression in a model of ischaemia support the idea that therapeutic manipulation of lipid metabolism in ischaemic cardiomyocytes may be beneficial. We hypothesise that therapeutic strategies in cardiac ischaemia and heart failure could be based on the fetal model of hypoxia protection, whereby modulation of metabolic substrate preserves oxygen at the expense of ATP production.

## Materials and Methods

All mouse experiments were carried out in compliance with institutional ethical and welfare standards and under Home Office regulation.

### Mouse Husbandry

Doxycycline 3 mg/kg was administered as described [9]. RNA was extracted from hearts using Trizol reagent (Invitrogen) according to the manufacturer's instructions. Complementary DNA was made using Superscripts 3 kits (Invitrogen). RTPCR was carried out on an Applied Biosystems 7000 analyser with SYBRGreen (Thermo Scientific), using *18s* RNA as a control. All PCR primers were purchased from Qiagen, or are listed in [9].

### High-Resolution Episcopic Microscopy

High-resolution episcopic microscopy was carried out as published [20].

### PCR Cloning of HSL Promoter and Luciferase Assay

A 0.9 kb fragment of the mouse HSL 5′ promoter was isolated by PCR using primers listed in Text S1. This was cloned into the pGL4 plasmid (Promega). Mutation of the e-box site was performed using a Quikchange SDM kit (Stratagene, Santa Clara). Luciferase activity was estimated using the Dual-Luciferase assay kit (Promega) and an Anthos Lucy spectrophotometer (Biochrom, Cambridge).

### Chromatin Immunoprecipitation

Chromatin was prepared from neonatal mouse hearts by previously published methods [50]. See Text S1 for details.

### Analysis of Acylcarnitines and Intact Lipids

Lipids were extracted using the methanol/chloroform/water method as described [51]. See Text S1 for details.

### Determination of Malonyl-CoA

Frozen hearts were homogenized in 6% perchloric acid to extract CoA esters, and homogenates were spun at 12,000×g for 5 min, 4°C. Malonyl-CoA concentration in the supernatant was measured using HPLC as described previously [47],[52].

### Lipid Uptake Assay

Hand1 transfected or control HL1 cells were incubated in serum HEPES buffered saline at 37°C for 10 min (two washes), then incubated in 5 µg/ml BODIPY-palmitate (Invitrogen) for 2 min at 37°C, washed with cold HBS, then imaged on a Zeiss confocal microscope. Fluorescence was quantified by ImageJ and normalized to cell number (10 high power fields per well, three wells per genotype).

### Glycogen Content Assay

Hearts were snap frozen immediately after sacrificing neonatal mice 2 h following caesarian section, before access to milk. Frozen hearts were ground in liquid nitrogen, and glycogen extracted and quantified enzymatically using the Abcam Glycogen Assay Kit, according to the manufacturer's instructions (Abcam, Cambridge, UK). Glucose was measured in a nonhydrolysed aliquot of each sample, and subtracted from the hydrolysed value, to give glycogen-derived glucose values. Samples were analysed on an Anthos Lucy Spectrophotometer (Biochrom, Cambridge).

### Stable Cell Lines

HL-1 cells were transfected with full-length Hand1pcDNA construct with FugeneHD (Invitrogen) accordingly to the manufacturer's instruction. At 48 h after transfection, growth medium was supplemented with 0.4 mg/ml G148 in order to select clones overexpressing Hand1. Individual clones were picked and expression levels of Hand1 were verified by qRT-PCR and Western blot. For oxygen consumption rate and mitochondrial function test, neomycin was removed several passages before.

### Oxygen Consumption

A Seahorse Bioscience Instrument was used to measure oxygen consumption rate as per manufacturer's instructions [53]. See Text S1 for details.

### Palmitate Oxidation

^3^H Labeled Palmitate (Sigma) oxidation was measured as previously described, incubating cells for 10 min [28].

### ATP, Phosphocreatine, and Malonyl coA Concentration

Hearts from adult HAND1 transgenic and control at 2 mo of age, following 1 mo of doxycycline dosage were collected under isoflurane anaesthesia and ventilation. Chest was opened and hearts were freeze-clamped in situ with aluminum clamps precooled in liquid nitrogen. Freeze dried hearts were extracted with 0.4 M perchloric acid, and extracts were neutralized with 2 M KOH. Metabolite levels were measured by HPLC using the procedure described by us previously. Malonyl-CoA concentration was measured in the same extracts using a previously published LC/MS procedure [54].

### Myocardial Ischaemia

Hearts were removed from adult mice and perfused on a Langendorff apparatus as previously described [30]. The ischaemia-reperfusion protocol consisted of 30 min stabilisation followed by 35 min global normothermic ischaemia and 30 min reperfusion. Global ischaemia was achieved by switching off the perfusion and immersing the heart in nonoxygenated buffer at 37°C.

At the end of the reperfusion period, hearts were perfused through the aortic cannula, with 1% prewarmed triphenyltetrazolium chloride (TTC) and then immersed in TTC at 37°C for 10 min. Then they were weighed and frozen at −20°C for 24 h. While still frozen, hearts were sliced from base to apex at a thickness of ∼≈1 mm. The slices were fixed in 10% formalin for 12 h to better define the boundaries between alive and dead tissue.

Heart slices were then photographed on a Perspex mounting block using a digital EsKape (Eskape, NY, USA) fixed camera. NIH Image 1.63 software was used to calculate the volumes of the whole heart and infarcted zones. The results were expressed as a I/R% of the dead tissue (I, infarct) developed in the whole heart (R, myocardium at risk) and presented as means ± standard error of the mean (SEM). The differences between groups were considered significant when *p*≤0.05.

### Metabolic Flux Analysis

See Text S1 for details.

## Supporting Information

Figure S1 **Structure of Hand1 overexpressing hearts immediately before birth is not significantly altered.** (A) Prenatal cardiac structure is not altered in induced *XMLC2-Hand1* e18 neonatal mouse hearts compared with control littermates. (B) Small heart size, but no gross structural defects in hearts removed from *XMLC2-Hand1* pups 4 h after caesarian section compared with control littermate. (i) shows eroded views though reconstructed episcopic sections at approximately the same point in one control and four *Hand1* up-regulating littermates 4 h after caesarian section. (ii) shows volume renders of the same datasets as (i), to show overall decrease in heart size (scale bar, 0.5 mm). (C, D) Immunohistochemical staining using antibody against cleaved caspase 3 on cryostat slices through Hand1 up-regulating and control neonatal hearts revealed no significant apoptosis in either group (*n* = 3 each group, 10 high-power fields examined) (control = XMLC, XMLC-Hand1 = Hand1 overexpressing hearts; red, cleaved caspase 3; blue, nuclear DAP1). Scale bar, 100 µm.
(TIF)

Figure S2 **Gene expression in neonatal XMLC-Hand1 hearts.** (A) Western blot to *Hand1* protein in stably transfected HL1 cell lines expressing Hand1, and ShRNA to Hand1. (B) RTPCR of chromatin immunoprecipitation data. Each bar represents the summary of three separate experiments. Error bars are standard deviation, and *p* values are two-tailed *t* test. (C) RTPCR of ChIP using anti-Hand1 antibody on chromatin prepared from e17.5 *Hand1* null hearts (α*MHC-Hand1^(fl/fl)^*). Lane 1 is ChIP using anti-Nkx2.5 antibody, assaying ANF chromatin, to show that overall chromatin quality in these samples is acceptable. (D) RTPCR showing no significant change in expression of mRNA encoding PPAR isoforms in neonatal p0.5 Hand1 up-regulating and control hearts (*n* = 4 each group). (E) RTPCR of RNA from p0.5 neonatal hearts from XMLC2 (Hand1 oe) and control pups, showing no significant difference in expression of RNA encoding glycolytic enzymes. (F) RTPCR of RNA from p0.5 neonatal hearts fromXMLC-Hand1 and control pups, showing no significant difference in expression of mRNA encoding PGC1*-α* or ERR*-α n* = 4 each group). (G) RTPCR of mRNA from p0.5 neonatal hearts from XMLC2 (Hand1 oe) and control pups, showing no significant difference in expression of mRNA encoding mitochondrial complex components (*n* = 4 each group). (H) RTPCR of mRNA from p0.5 neonatal hearts from αMHC-Cre::VHL^(fl/fl)^ and control pups, showing no significant difference in expression of mRNA encoding PGC1*-α* or ERR*-α n* = 6 each group). (I) RTPCR of mRNA Hl1 cells stably transected with vectors encoding Hand1 or shRNA to Hand1 showing down-regulation of fatty acid metabolising genes in Hand1 up-regulation, and up-regulation of many of these genes in Hand1 knockdown. (J) Western blot of protein extracts from HL1 cells stably transfected with Hand1 overexpression vector, empty vector, and untransfected. We found no difference in Hand1, PGC1 α, or Hand2 between empty vector and untransfected lines.
(TIF)

Figure S3 **Oxygen consumption in HL1 cells.** Absolute values of oxygen consumption for Figure 5C.
(TIF)

Movie S1 **XMLC-Hand1 neonatal mice suffer respiratory distress.** Cyanosis and respiratory distress in a neonatal (p0.5) *XMLC2-Hand1* pup, left of screen, with a control pup on the right for comparison.
(MOV)

Table S1 **Gene expression changes in XMLC-Hand1 neonatal hearts.** Affymetrix analysis of differential RNA expression of three hearts from p0.5 *XMLCrTTA::tetHand1* pups and three controls, using Genechip 430B. All hits showing significance *p*>0.05 shown.
(XLSX)

Table S2 **Gene ontology signal of changes in XMLC-Hand1 neonatal hearts.** Gene ontology analysis of Affymetrix gene expression hits in *Hand1* prolonging neonatal hearts shows an overrepresentation of genes tagged with metabolic function (A). Functional Annotation Tag (FAT) search reveals overrepresentation of fatty acid metabolic process tags. (B) Gene ontology pathway overrepresentation analysis.
(DOC)

Table S3 **Acylcarnitine expression changes in XMLC-Hand1 neonatal hearts.** Acylcarnitine species in 6 *Hand1* up-regulating neonatal (p0.5) and six control hearts.
(XLSX)

Table S4 **XMLC-Hand1 neonatal hearts display a decrease in high energy phosphate content.** High energy phosphate metabolites in adult *XMLCrTTA::tetHand1* up-regulating hearts and controls, showing reduced phosphocreatine/creatine and Phosphocreatine/ATP ratios. Animals were 8 wk old and had been induced with doxycycline for 4 wk.
(XLS)

Text S1 **Supplemental materials and methods.**
(DOCX)

## Abbreviations

ACC: acetyl coA carboxylase
ACSL: acyl coA synthase long chain 1
ATGL: adipose triglyceride lipase
ChIP: chromatin immunoprecipitation
CPT: carnitine palmitoyl transferase
FABP: fatty acid binding protein
FATP: fatty acid transport protein
HIF: Hypoxia Inducible factor
HSL: hormone sensitive lipase
MCD: malonyl coA decarboxylase
MHC: myosin heavy chain promoter
XMLC2: *Xenopus* myosin light chain 2 promoter
