# Correction: Hypoxic Regulation of *Hand1* Controls the Fetal-Neonatal Switch in Cardiac Metabolism

**DOI:** 10.1371/annotation/a9a7f37a-3fa7-4f7f-8310-1339bf5a666e

**Published:** 2013-12-19

**Authors:** Ross A. Breckenridge, Izabela Piotrowska, Keat-Eng Ng, Timothy J. Ragan, James A. West, Surendra Kotecha, Norma Towers, Michael Bennett, Petra C. Kienesberger, Ryszard T. Smolenski, Hillary K. Siddall, John L. Offer, Mihaela M. Mocanu, Derek M. Yelon, Jason R. B. Dyck, Jules L. Griffin, Andrey Y. Abramov, Alex P. Gould, Timothy J. Mohun

The supplemental methods file included as part of this article was submitted with details missing regarding the protocols and antibodies used for the western blot and ChIP assays. An amended version of this file has now been included in the supporting information for this article, available via 
