# Supplementary figures and images for: Hypoxic Regulation of Hand1 Controls the Fetal-Neonatal Switch in Cardiac Metabolism
Source: PLoS Biol. 2013 Sep 24;11(9):e1001666. doi: 10.1371/journal.pbio.1001666 (PMC3782421; doi:10.1371/journal.pbio.1001666)

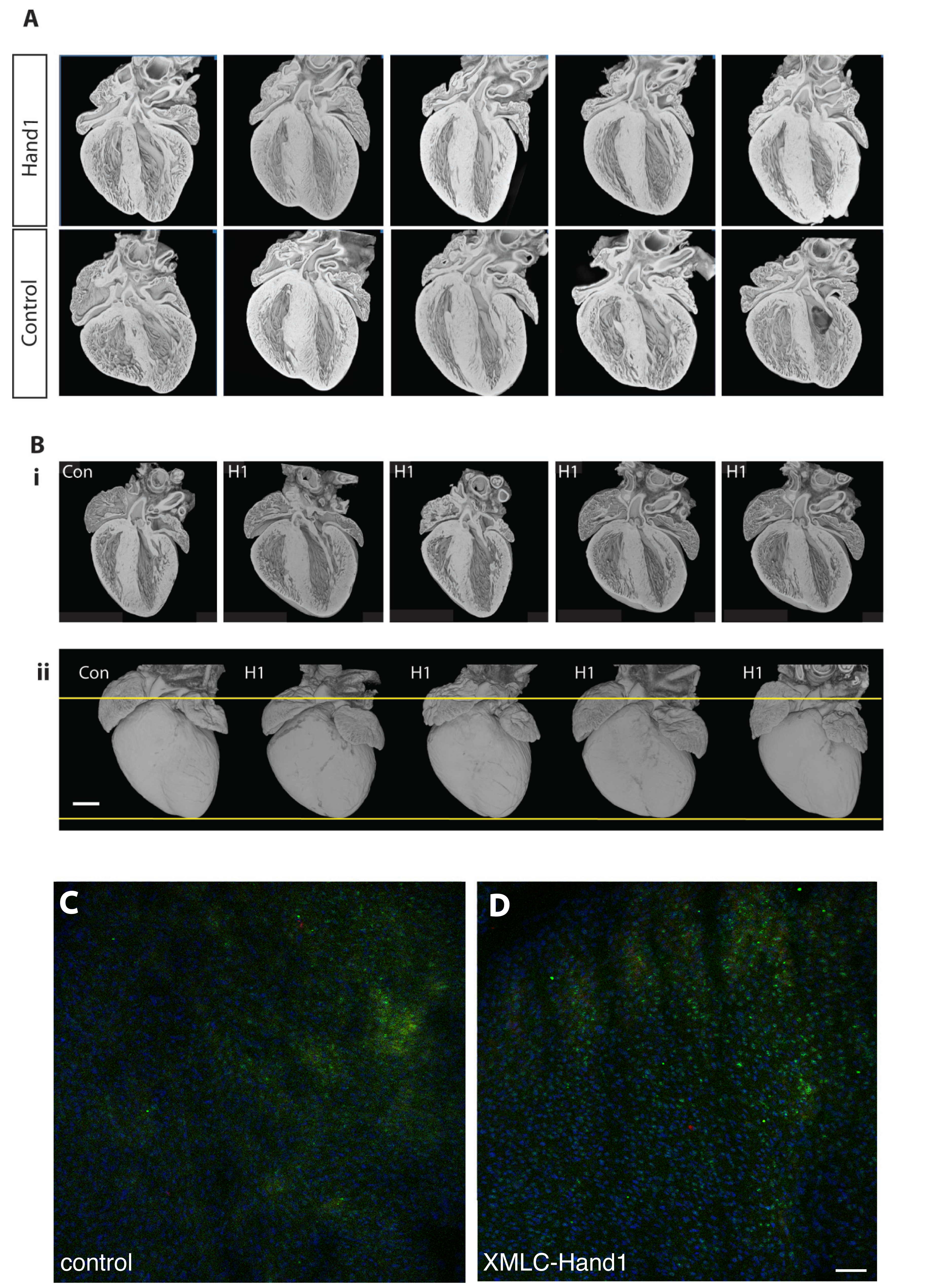

Supplement: Figure S1 — Structure of Hand1 overexpressing hearts immediately before birth is not significantly altered. (A) Prenatal cardiac structure is not altered in induced XMLC2-Hand1 e18 neonatal mouse hearts compared with control littermates. (B) Small heart size, but no gross structural defects in hearts removed from XMLC2-Hand1 pups 4 h after caesarian section compared with control littermate. (i) shows eroded views though reconstructed episcopic sections at approximately the same point in one control and four Hand1 up-regulating littermates 4 h after caesarian section. (ii) shows volume renders of the same datasets as (i), to show overall decrease in heart size (scale bar, 0.5 mm). (C, D) Immunohistochemical staining using antibody against cleaved caspase 3 on cryostat slices through Hand1 up-regulating and control neonatal hearts revealed no significant apoptosis in either group (n = 3 each group, 10 high-power fields examined) (control = XMLC, XMLC-Hand1 = Hand1 overexpressing hearts; red, cleaved caspase 3; blue, nuclear DAP1). Scale bar, 100 µm. (TIF) [file pbio.1001666.s001.tif]

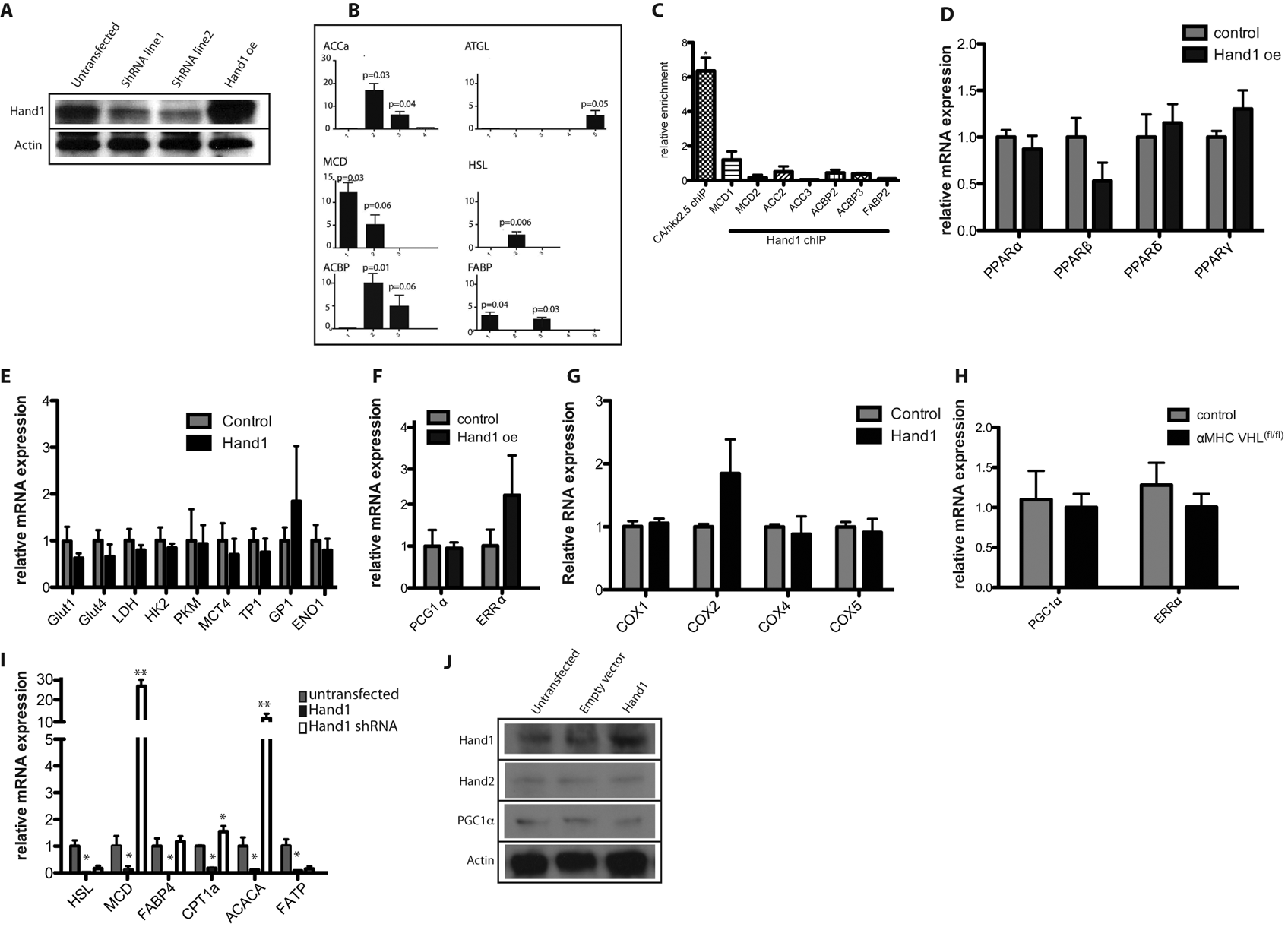

Supplement: Figure S2 — Gene expression in neonatal XMLC-Hand1 hearts. (A) Western blot to Hand1 protein in stably transfected HL1 cell lines expressing Hand1, and ShRNA to Hand1. (B) RTPCR of chromatin immunoprecipitation data. Each bar represents the summary of three separate experiments. Error bars are standard deviation, and p values are two-tailed t test. (C) RTPCR of ChIP using anti-Hand1 antibody on chromatin prepared from e17.5 Hand1 null hearts (αMHC-Hand1(fl/fl)). Lane 1 is ChIP using anti-Nkx2.5 antibody, assaying ANF chromatin, to show that overall chromatin quality in these samples is acceptable. (D) RTPCR showing no significant change in expression of mRNA encoding PPAR isoforms in neonatal p0.5 Hand1 up-regulating and control hearts (n = 4 each group). (E) RTPCR of RNA from p0.5 neonatal hearts from XMLC2 (Hand1 oe) and control pups, showing no significant difference in expression of RNA encoding glycolytic enzymes. (F) RTPCR of RNA from p0.5 neonatal hearts fromXMLC-Hand1 and control pups, showing no significant difference in expression of mRNA encoding PGC1-α or ERR-α n = 4 each group). (G) RTPCR of mRNA from p0.5 neonatal hearts from XMLC2 (Hand1 oe) and control pups, showing no significant difference in expression of mRNA encoding mitochondrial complex components (n = 4 each group). (H) RTPCR of mRNA from p0.5 neonatal hearts from αMHC-Cre::VHL(fl/fl) and control pups, showing no significant difference in expression of mRNA encoding PGC1-α or ERR-α n = 6 each group). (I) RTPCR of mRNA Hl1 cells stably transected with vectors encoding Hand1 or shRNA to Hand1 showing down-regulation of fatty acid metabolising genes in Hand1 up-regulation, and up-regulation of many of these genes in Hand1 knockdown. (J) Western blot of protein extracts from HL1 cells stably transfected with Hand1 overexpression vector, empty vector, and untransfected. We found no difference in Hand1, PGC1 α, or Hand2 between empty vector and untransfected lines. (TIF) [file pbio.1001666.s002.tif]

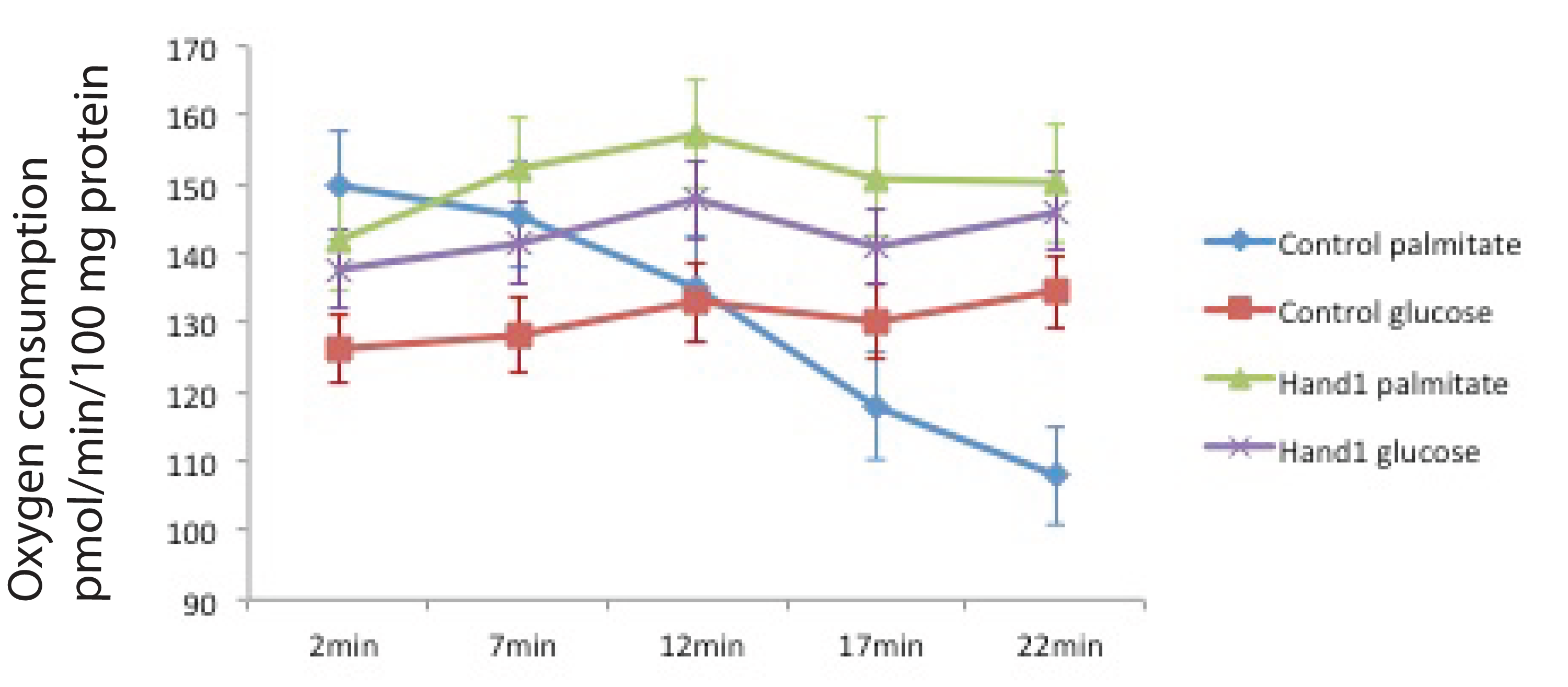

Supplement: Figure S3 — Oxygen consumption in HL1 cells. Absolute values of oxygen consumption for Figure 5C. (TIF) [file pbio.1001666.s003.tif]
