# Supplementary material for: Hypoxic Regulation of Hand1 Controls the Fetal-Neonatal Switch in Cardiac Metabolism
Source: PLoS Biol. 2013 Sep 24;11(9):e1001666. doi: 10.1371/journal.pbio.1001666 (PMC3782421; doi:10.1371/journal.pbio.1001666)
Supplement: Table S2 — Gene ontology signal of changes in XMLC-Hand1 neonatal hearts. Gene ontology analysis of Affymetrix gene expression hits in Hand1 prolonging neonatal hearts shows an overrepresentation of genes tagged with metabolic function (A). Functional Annotation Tag (FAT) search reveals overrepresentation of fatty acid metabolic process tags. (B) Gene ontology pathway overrepresentation analysis. (DOC) [file pbio.1001666.s006.doc]

1. GO Functional annotation search level 2

| **GO term** | **Gene count** | **% of genes** | **P value** |
| --- | --- | --- | --- |
| **primary metabolic process** | 245 | 39.7 | 5.8E-5 |
| **cellular metabolic process** | 230 | 37.3 | 2.0E-4 |
| **macromolecule metabolic process** | 203 | 32.9 | 3.9E-4 |
| **Regulation of metabolic process** | 127 | 20.6 | 4.6E-4 |
| **Macromolecule organisation** | 49 | 7.9 | 5.8E-4 |
| **Establishment of protein localisation** | 36 | 5.8 | 3.1E-3 |
| **Biosynthetic process** | 125 | 20.3 | 20.3E-3 |
| **Positive regulation of cellular process** | 61 | 9.9 | 5.4E-3 |
| **Organelle localisation** | 51 | 8.3 | 7.6E-3 |
| **Cellular response to a stimulus** | 30 | 4.9 | 9.0E-3 |

1. GO FAT search

| **GO term** | **Gene count** | **% of genes** | **P value** |
| --- | --- | --- | --- |
| **Cellular protein localisation** | 22 | 3.6 | 9.6E-4 |
| **Cellular macromolecule localisation** | 22 | 3.6 | 1.0E-3 |
| **Fatty acid metabolic process** | 16 | 2.6 | 1.1E-3 |
| **transcription** | 82 | 13.3 | 1.2E-3 |
| **Protein localisation** | 41 | 6.6 | 2.0E-3 |
| **Intracellular signaling** | 47 | 7.6 | 2.8E-3 |
| **Protein transport** | 36 | 5.8 | 3.0E-3 |
| **Establishment of protein localisation** | 38 | 5.8 | 3.5E-3 |
| **Negative regulation of molecular function** | 12 | 1.9 | 4.3E-3 |
| **Intracellular protein transport** | 19 | 3.1 | 4.8E-3 |
| **Fatty acid biosynthetic process** | 9 | 1.5 | 5.0E-3 |
