# Supplementary material for: Correction: Hypoxic Regulation of Hand1 Controls the Fetal-Neonatal Switch in Cardiac Metabolism
Source: PLoS Biol. 2013 Dec 19;11(12):10.1371/annotation/a9a7f37a-3fa7-4f7f-8310-1339bf5a666e. doi: 10.1371/annotation/a9a7f37a-3fa7-4f7f-8310-1339bf5a666e (PMC3868671; doi:10.1371/annotation/a9a7f37a-3fa7-4f7f-8310-1339bf5a666e)
Supplement: Supplementary file 1 [file pbio.a9a7f37a-3fa7-4f7f-8310-1339bf5a666e.s001.docx]

**Supporting Information**

**Supplemental Experimental Procedures**

**Chromatin Immunoprecipitation**

Chromatin was prepared from neonatal mouse hearts by previously published methods ([Depre and Vatner, 2005](#_ENREF_3)). Tissue was fixed for 3 hours at room temperature in buffer (50mM HEPES pH7.9, 1mM EDTA, 1mM EGTA, 100mM NaCl, 0.07% butryric acid) containing 1.8% formaldehyde. The tissue homogenised using an Ultra-Turrax, T25 basic (IKA-Werke), and pelleted at low speed. Samples were washed twice with ice-cold PBS with freshly added EDTA-free protease inhibitors (PI) (Roche). Samples were resuspended in 1.5ml lysis buffer (25mM Tris pH 7.5, 150mM NaCl, 1% Triton X100, 1% SDS, 2.5mM Sodium deoxycholate, PI) and transferred to RNase-free non-stick microfuge tubes (Ambion). Samples sonicated for 15 x 30 seconds on ice using a Branson Digital Sonicator with a 2.5mm stepped probe tip. Samples were spun at 15K using a bench top centrifuge for 15 minutes at 4ºC. The supernatant was then pre-blocked with protein A/G sepharose (Perbio), pre-treated with BSA (Biorad) and Poly (dI-dC)-poly (dI-dC) (GE Healthcare). A proportion of the sample was kept at this stage as an input control. 150μl of chromatin was diluted 10 fold in ChIP dilution buffer (16.7mM Tris pH7.5, 0.01% SDS, 1.1% Triton, 1.2mM EDTA, 167mM NaCl) and 4μg of either HIF1a (Novus Biochem NB100-105) or Hand1 (eHand1 C-19, Santa Cruz) antibody was added and incubated overnight at 4ºC with rotation. The antibody:protein:DNA complexes were captured using pre-blocked protein A/G sepharose for 2 hours rotating at 4ºC. The beads were then spun down and washed twice in wash buffer A (10mM HEPES pH 7.6, 1mM EDTA, 0.5mM EGTA, 0.25% Triton X100) and twice with wash buffer B (10mM HEPES pH7.6, 200mM NaCl, 1mM EDTA, 0.5mM EGTA, 0.01% Triton X100. After extensive washing, the beads were resuspended in TE and cross-links reversed overnight at 65ºC. Samples were adjusted to 0.1% SDS, digested with 10μg Proteinase K (Roche) at 50ºC for 3 hours and extracted twice with phenol/chloroform. Samples were ethanol precipitated with glycogen carrier (Ambion) and resuspended in 100μl TE. RTPCR was carried out using SYBRGreen (Thermo Scientific).

**Western Blotting**

Samples were boiled in SDS for 2 minutes, and loaded onto pre-cast 10% acrylamide gels (Bio-Rad). Blots were blocked in 4% BSA in TBST, and autoradiographs generated using ECL Prime reagent (GE). HRP conjugated secondary antibodies were all purchased from GE. Healthcare, and used at 1/2000 dilution (In 4% BSA TBST)

Antibodies used for Western blots;

HIF1α Abcam Ab82832. Used at 1/2000 dilution in 4% BSA TBST

PGC1 α Abcam Ab54481. Used at 1/2000 dilution in 4% BSA TBST

Hand1 in-house sheep anti Hand1 antibody as previously described ([Breckenridge et al., 2009](#_ENREF_1)). Used at 1/500 dilution in 4% BSA TBST

Hand2 Abcam Ab56590. Used at 1/2000 dilution in 4% BSA TBST

Actin Sigma A5060. Used at 1/200 dilution in 4% BSA TBST

**Analysis of acylcarnitines and intact lipids**

Lipids were extracted using the methanol/chloroform/water method as described ([Le Belle et al., 2002](#_ENREF_4)).

One fifth of the organic fraction of each sample was derivatised with 100µl of 3M butanolic HCL (Sigma-Aldrich, Louis, Missouri, USA) for 15min at 60⁰C. The samples were dried under nitrogen and reconstituted in 500µl of acetonitrile containing a derivatised internal standard mixture (Cambridge Isotope Laboratories, Inc., Andover, Massachusetts, USA). Samples were analysed on a Quattro™ Premier XE triple quadrupolar mass spectrometer (Waters Ltd., Herfordshire, UK) using ESI with a parent scan in positive ion mode for a common loss of +85 Da with the following optimisation parameters: capillary voltage 3.5 kV, cone voltage 35V, collision energy 25V, source temperature 110⁰C and desolvation temperature 300⁰C. Samples were introduced by direct infusion with a run time of 5min and a flow rate of 10µl/min that increased to 100µl/min via a linear gradient over the last minute. The mobile phase consisted of 1:1 acetonitrile: *iso*-propanol with 0.2% formic acid (Sigma-Aldrich, Louis, Missouri, USA.). Data were processed using the Neolnyx™ package software addition to the Masslynx™ program (Waters Ltd., Herfordshire, UK.).

One fifth of the organic fraction was used for analysis. A pool sample was prepared by combining 5µl of each sample. The samples were analysed using a QToF Ultima mass spectrometer (Waters Ltd., Hertfordshire, UK.) in conjunction with an Acquity UPLC system (Waters Ltd., Hertfordshire, UK.). Chromatographic separation of the compounds was carried out using a 1.7µm Bridged Ethyl Hybrid (BEH) C8 UPLC column (Waters Ltd.) set at a temperature of 65⁰C using a binary solvent system comprising A: HPLC grade water, 0.1% formic acid and 1% 1M ammonium acetate and B: 5:2 acetonitrile: *iso*-propanol, 0.1% formic acid and 1% 1M ammonium acetate ([Roberts et al., 2009](#_ENREF_6); [Sysi-Aho et al., 2011](#_ENREF_7)). A linear gradient was employed, at a flow rate of 600µl/min, of 60-100% mobile phase B over the first 8 minutes, 100% B for a further 2 minutes and then re-equilibration of the column at 60% B for the remaining 2 minutes of the run. The mass spectrometer was operated in positive ion mode using ESI with the following parameters: capillary voltage 3 kV, cone voltage 50V, source temperature 80⁰C and desolvation temperature 250⁰C and was set to scan in MS scan mode between 80 and 1500 m/z over a 12min run time.

Data was collated and normalised in Excel™ (Microsoft). Acyl carnitine data were imported into SIMCA-P+ version 12.0 (Umetrics, Umeå, Sweden) for multivariate partial least squares discriminant (PLS-DA) statistical analysis where unit variance (UV) scaling was used. To assess the reliability and significance of our PLS-DA model we calculate values Q^2^ and R^2^. R^2^ is a value from 0 to 1 and gives a measure of how well fitted the data is to the components in our scores plot. Q^2^ is a value from 0 to 1 and displays the prediction for our scores plot, that is, how reliable a separation between two data sets is. Metabolite changes responsible for clustering or regression trends within the pattern recognition models were identified by examining the corresponding loadings plot.

**Oxygen consumption**

A Seahorse Bioscience Instrument was used to measure oxygen consumption rate as per manufacturers instructions ([Wu et al., 2007](#_ENREF_8)). 6x105 cells (Hand1 overexpressing or control cells) per well were plated on 24 well XF plate 24 hours before assay and grown in standard Claycomb medium as previously described ([Claycomb et al., 1998](#_ENREF_2)). Transfections were carried out using lipofectamine (Quiagen) and shRNA constructs to Hand1 were purchased from Quiagen. In order to measure oxygen consumption in response to 11mM glucose or 20mM palmitate, cells were starved for 3 hours prior to the assay. For oxygen consumption rate measurement, growth medium was replaced with XF assay medium pH7.4. To asses mitochondrial function 0.5µM oligomycin, 1 µM FCCP and 1µM Rotanone were used. For inhibition of beta-oxidation 50µM Etomoxir was injected. Results were normalized to protein concentration/well. Palmitate-BSA conjugate was generated as described previously ([Pike et al., 2011](#_ENREF_5)).

PCR primer sequences for chromatin immunoprecipitation

| FATF | AAAGACTGCCCGAGTGCAAA |
| --- | --- |
| FATR | TGACCAAGAATCTTCCTCAAGC |
| FAB1F | TTGCATATCTGCCCTCATGGA |
| FAB1R | AGAACTTACCAGCAGGCCAGCT |
| FAB2F | GCCCTGATTTACAGATACCCCC |
| FAB2R | AGATGACAACCGCCACAACAC |
| FAB3 F | ATGTTCCTTGCTCCGTGGATC |
| FAB3R | TCTCTTGGAACTGGTAGCGCAG |
| FAB4F | GCTCTACAGCACGTCTAACCCA |
| FAB4R | TGGAAACGTATGCTGATCCCTA |
| FAB5F | TTCCTGAGCAATGAGCAGAATG |
| FAB5R | TCAAAGAAACCTAGCTGCCCC |
| LIPE1F | TGAGCCTCTTCCTAGTCCTAATCC |
| LIPE1R | CACTGCTGGTCCCCACTTCT |
| LIPE2F | GGACCACTCATCTAGCTCACCTAGA |
| LIPE2R | AAAATGACCAGTGACCCTTCTCA |
| LIPE3F | TGAGCCTCTTCCTAGTCCTAATCC |
| LIPE3R | CACTGCTGGTCCCCACTTCT |
| ATGL2F | AAGTTCTTTGCGGTGTATGTCAAG |
| ATGL2R | CAACAATTCAACCTGAAGATCTCTGA |
| ATGL3F | AAGCTGAAGCCTGGGAATCC |
| ATGL3R | CCGCCCCTTCGTGACA |
| ATGL4F | TGAGACCAAGTAGACCCCACAA |
| ATLG4R | AGCTGCTCACTTGGGTTTTAGC |
| ATGL5F | CACAAACTCCTGGTACCCAGATC |
| ATGL5R | GGCAGCCCCTGGACTCA |
| ACACA1F | CGAACTCAGAAATCCACCTGC |
| ACACA1R | TCGAAACAATGCCACACACA |
| ACACA2F | CCGGAAGAGGATGTTGGATTC |
| ACACA2R | AAGAGCACTGCTCTTCCAGAGG |
| ACACA3F | GTTCAGAAGAGGATGGCCAGTC |
| ACACA3R | GCTTTTGCAGAAGACCACAGCT |
| ACACA4F | TGTATGTCCGTGCACCACATG |
| ACACA4R | AATTTGCTCCCAGCACCTGAC |
| DBI1F | AGCCCCTCTCAACATCTGAGA |
| DBI1R | GGATGACTTTCCTACCATGCAG |
| DBI2F | AGAAACTCTGCGTGCTGGCTA |
| DBI2R | TTGTCAGGCAGGTCAGTATCGT |
| DBI3F | AGGCATTTCACACCTGCTCAG |
| DBI3R | TGGAGCTTGTCCTCGTGGTTA |
| DBI4F | TCTGTTTGTCCTCTGCTGCGT |
| DBI4R | CCATCGAGCCTTGGTTAAGTG |
| DBI5F | ACCCCATTTCACGCGTTCT |
| DBI5R | CATTCTCCGTCAGGGCAGTT |
| MLYCD1F | AAAAAAACGAAACGCGTCCC |
| MLYCD1R | AGCAGCCAATCAGAAGTCGGA |
| MLYCD2F | GTCGGCTTATTGAAACCAGCA |
| MLYCD2R | AAACCCAGTTCAGTTCCCACA |

Primers for HIF chIP of Hand1 promoter

P1F TGAGTGTCCATTGTCCTTGTGG

P1R TGCCTCCTGCATAGTTTCCAAA

P2F GATTTTCATGCAGGTGGCGA

P2R AAGGCATCTCTGTGGCAGGACT

P3F CTGGCTACCAGTTACATCGCCT

P3R CCACCATCCGTCTTTTTGAGTT

P4F CTGGCTACCAGTTACATCGCCT

P4R CCACCATCCGTCTTTTTGAGTT

P5F TTAAAAGCCTGGTCTCCCACG

P5R AACCTTGTGTTGCTCCCCATC

Primers for PCR cloning of HSL promoter fragment

5’ CCTAGATCTTCCAGAGGTCCTGAGTTCAATTCCCA

3’ AGAGAATTCGGCTTCATCCTTCTGCTCCCTACCCTTTTCAGGCTTC

Primers for Site directed mutagenesis of LIPE promoter fragment

5’ TAGCTCACCTAGAATCATCCCCGAAGAAACAGAAGACTAAGA

3’ TCTTAGTCTTCTGTTTCTTCGGGGCTGATTCTAGGTGAGCTA

**Mitochondrial membrane potential analysis**

For measurements of ΔΨ_m_, cells plated on 22mm glass coverslips were loaded for 30 min at room temperature with 25 nM tetramethylrodamine methylester (TMRM; Invitrogen) in a HEPES buffered saline solution (HBSS) composed of 156 mM NaCl, 3 mM KCl, 2 mM MgSO_4_, 1.25 mM KH_2_PO_4_, 2mM CaCl_2_, 10 mM glucose and 10 mM HEPES; pH adjusted to 7.35 with NaOH. The dye remained present in the media at the time of recording. Confocal images were obtained using a Zeiss 710 VIS CLSM equipped with a META detection system and a 40x oil immersion objective. TMRM was excited using the 560 nm laser line and fluorescence was measured above 580 nm. For basal ΔΨ_m_ measurements, Z-stack images were obtained by confocal microscopy and analysed using Zeiss software.

**Measurement of NADH redox index**

NADH autofluorescence was measured using an epifluorescence inverted microscope equipped with a 20x fluorite objective. Excitation light at a wavelength of 350nm was provided by a Xenon arc lamp, the beam passing through a monochromator (Cairn Research, Kent, UK). Emitted fluorescence light was reflected through a 455nm long-pass filter to a cooled CCD camera (Retiga, QImaging, Canada) and digitised to 12 bit resolution. Imaging data were collected and analysed using software from Andor (Belfast, UK).

**References**

Breckenridge, R.A., Zuberi, Z., Gomes, J., Orford, R., Dupays, L., Felkin, L.E., Clark, J.E., Magee, A.I., Ehler, E., Birks, E.J.*, et al.* (2009). Overexpression of the transcription factor Hand1 causes predisposition towards arrhythmia in mice. J Mol Cell Cardiol *47*, 133-141.

Claycomb, W.C., Lanson, N.A., Jr., Stallworth, B.S., Egeland, D.B., Delcarpio, J.B., Bahinski, A., and Izzo, N.J., Jr. (1998). HL-1 cells: a cardiac muscle cell line that contracts and retains phenotypic characteristics of the adult cardiomyocyte. Proc Natl Acad Sci U S A *95*, 2979-2984.

Depre, C., and Vatner, S.F. (2005). Mechanisms of cell survival in myocardial hibernation. Trends in cardiovascular medicine *15*, 101-110.

Le Belle, J.E., Harris, N.G., Williams, S.R., and Bhakoo, K.K. (2002). A comparison of cell and tissue extraction techniques using high-resolution 1H-NMR spectroscopy. NMR in biomedicine *15*, 37-44.

Pike, L.S., Smift, A.L., Croteau, N.J., Ferrick, D.A., and Wu, M. (2011). Inhibition of fatty acid oxidation by etomoxir impairs NADPH production and increases reactive oxygen species resulting in ATP depletion and cell death in human glioblastoma cells. Biochimica et biophysica acta *1807*, 726-734.

Roberts, L.D., Hassall, D.G., Winegar, D.A., Haselden, J.N., Nicholls, A.W., and Griffin, J.L. (2009). Increased hepatic oxidative metabolism distinguishes the action of Peroxisome proliferator-activated receptor delta from Peroxisome proliferator-activated receptor gamma in the ob/ob mouse. Genome medicine *1*, 115.

Sysi-Aho, M., Ermolov, A., Gopalacharyulu, P.V., Tripathi, A., Seppanen-Laakso, T., Maukonen, J., Mattila, I., Ruohonen, S.T., Vahatalo, L., Yetukuri, L.*, et al.* (2011). Metabolic regulation in progression to autoimmune diabetes. PLoS computational biology *7*, e1002257.

Wu, M., Neilson, A., Swift, A.L., Moran, R., Tamagnine, J., Parslow, D., Armistead, S., Lemire, K., Orrell, J., Teich, J.*, et al.* (2007). Multiparameter metabolic analysis reveals a close link between attenuated mitochondrial bioenergetic function and enhanced glycolysis dependency in human tumor cells. Am J Physiol Cell Physiol *292*, C125-136.
